# Whole Genome Association Studies of Residual Feed Intake and Related Traits in the Pig

**DOI:** 10.1371/journal.pone.0061756

**Published:** 2013-06-26

**Authors:** Suneel K. Onteru, Danielle M. Gorbach, Jennifer M. Young, Dorian J. Garrick, Jack C. M. Dekkers, Max F. Rothschild

**Affiliations:** Department of Animal Science and Center for Integrated Animal Genomics, Iowa State University, Ames, Iowa, United States of America; Auburn University, United States of America

## Abstract

**Background:**

Residual feed intake (RFI), a measure of feed efficiency, is the difference between observed feed intake and the expected feed requirement predicted from growth and maintenance. Pigs with low RFI have reduced feed costs without compromising their growth. Identification of genes or genetic markers associated with RFI will be useful for marker-assisted selection at an early age of animals with improved feed efficiency.

**Methodology/Principal findings:**

Whole genome association studies (WGAS) for RFI, average daily feed intake (ADFI), average daily gain (ADG), back fat (BF) and loin muscle area (LMA) were performed on 1,400 pigs from the divergently selected ISU-RFI lines, using the Illumina PorcineSNP60 BeadChip. Various statistical methods were applied to find SNPs and genomic regions associated with the traits, including a Bayesian approach using GenSel software, and frequentist approaches such as allele frequency differences between lines, single SNP and haplotype analyses using PLINK software. Single SNP and haplotype analyses showed no significant associations (except for LMA) after genomic control and FDR. Bayesian analyses found at least 2 associations for each trait at a false positive probability of 0.5. At generation 8, the RFI selection lines mainly differed in allele frequencies for SNPs near (<0.05 Mb) genes that regulate insulin release and leptin functions. The Bayesian approach identified associations of genomic regions containing insulin release genes (e.g., *GLP1R, CDKAL, SGMS1*) with RFI and ADFI, of regions with energy homeostasis (e.g., *MC4R*, *PGM1*, *GPR81*) and muscle growth related genes (e.g., *TGFB1*) with ADG, and of fat metabolism genes (e.g., *ACOXL*, *AEBP1*) with BF. Specifically, a very highly significantly associated QTL for LMA on SSC7 with skeletal myogenesis genes (e.g., *KLHL31*) was identified for subsequent fine mapping.

**Conclusions/significance:**

Important genomic regions associated with RFI related traits were identified for future validation studies prior to their incorporation in marker-assisted selection programs.

## Introduction

Feed is the biggest variable cost in most livestock production systems, including pig farms. Profitability of pork production depends on feed efficiency, which can be measured by the feed to gain ratio. One estimate showed that reducing the feed to gain ratio from 2.75 to 2.45 could save swine producers in the US $500 million dollars annually [1]. Another measure of feed efficiency is residual feed intake (RFI), which is the difference between a pig's actual feed intake and its expected feed intake requirement predicted based on the animal's growth and maintenance. Generally in pigs, the predicted feed requirement is determined based on metabolic body weight, average daily gain (ADG) and back fat (BF) [2], [3]. Being a residual, RFI is phenotypically independent from metabolic weight, ADG and BF and represents differences in feed efficiency (feed to gain) that are independent of weight, ADG, and BF. Pig studies have shown RFI to be moderately heritable (0.18 to 0.41) [3], [4], [5], [6], [7], [8], [9]. Consequently, RFI is a candidate trait for selection to improve feed efficiency, along with selection for increased growth rate and reduced BF. Although RFI is an important trait, measurement of the phenotype requires collection of average daily feed intake (ADFI), which is expensive and difficult. Hence, identification of genes or markers associated with RFI and its related traits will be useful in applying marker-assisted selection for feed efficiency at an early age with lower cost than can be achieved measuring ADFI. To develop a resource population for elucidating the biological and genetic aspects of RFI, Iowa State University (ISU) has been developing selection lines for RFI for over a decade [2], [10].

Whole genome association studies (WGAS) using high-density SNP genotypes are efficient tools to identify genes or genomic regions that explain variation in livestock traits. WGAS studies can be based on different statistical methods, including frequentist and Bayesian approaches [11], [12], [13]. Several WGAS were performed for RFI in cattle [14], [15], [16], [17], [18] using frequentist approaches. One study [19] used Bayesian approaches to estimate the accuracies of estimated breeding values for RFI in beef cattle. However, few WGAS have been undertaken for RFI in pigs. The present study used both frequentist and Bayesian approaches to analyze the whole genome for associations with RFI and related traits such as ADFI, ADG and BF, as well as for a production trait, loin muscle area (LMA), in the ISU RFI pig selection lines.

## Materials and Methods

### Population

**Figure 1:**
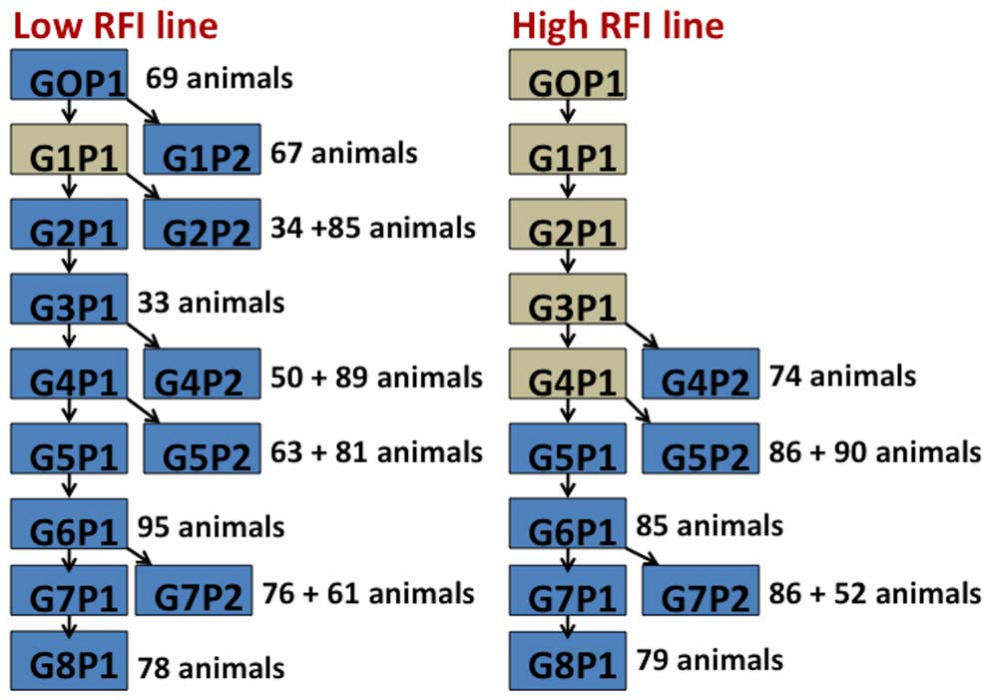
Population sampled. The animals in blue boxes were phenotyped and genotyped and included in the analyses. G: Generation; P: Parity. Please refer to references [2], [10] for the population structure, which defines generation and parity.

Animal care guidelines were followed according to the Institutional Animal Care and Use Committee (IACUC) at ISU (IACUC permit number 11-1-4996-S). Data on a total of 1433 Yorkshire pigs were included in this study. Starting from split litters from purebred Yorkshires in generation 0, these animals belonged to generations 0 to 8 of two selection lines for RFI [2], [10]. The numbers of animals from each generation are depicted in Figure 1. The low RFI line was selected for decreased RFI (increased efficiency) for all 8 generations. The high RFI line was randomly mated until generation 4 and was selected for increased RFI (decreased efficiency) starting in generation 5. Selection is on-going and has been successful [2], demonstrating a difference in RFI of 117 g/day between the two lines in generation 8. This RFI difference was obtained from the current data used for this study.

### Phenotypes

Daily feed intake was collected on each animal starting around 90 days of age (on-test) using electronic Feed Intake Recording Equipment (FIRE, Osborne, KS, USA) feeders donated by PIC (Hendersonville, TN, USA) and Newsham Choice Genetics (West Des Moines, IA, USA). Pigs were put on-test in 2 to 3 groups based on age. Sixteen pigs of a similar age and weight were penned together in one of 12 pens, which contained either a FIRE feeder or similar feeder. Prior to generation 7, only 6 pens contained a FIRE feeder with the other 6 pens containing a FIRE-like feeder. Pigs were switched every two weeks between a pen with a FIRE feeder and one with a FIRE-like feeder. Starting with generation 7, all 12 pens contained FIRE feeders. Body weights were collected every 2 weeks during the testing period. Upon reaching approximately 115 kg, 10^th^-rib BF and LMA were evaluated with an Aloka ultrasound machine (Corometrics Medical Systems, Inc., Wallingford, CT, USA). Among 1433 animals genotyped, 1417 had ADFI data, 1418 had ADG data, 1412 had BF data, and 1410 animals had LMA and RFI phenotypes.

The ADG phenotype was calculated by regressing weights on days on test with ADG being equal to the slope of the regression line. Feed intake data were edited using methods developed by [20] to account for missing data. ADFI was then calculated using quadratic random regression of feed intake data from on-test day to off-test day. Finally, RFI was computed similar to [2], where a single trait animal model was used to analyze ADFI with adjustments for metabolic mid weight, ADG, BF, weight at on-test, and weight at off-test. The following equation was used to compute RFI for each pig:
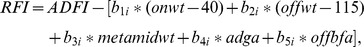
where i represents each combination of generation and line; onwt and offwt are the weight at on-test and off-test, respectively; metamidwt is the average weight of the pig while on test raised to the 0.75 power, which represents the metabolic mid-weight; adga is ADG adjusted to testing from 90 to 180 days of age; and offbfa is off-test BF adjusted to 115 kg of body weight. The regression coefficients were computed with a model that included random effects of animal (genetic), dam, and pen within on-test group, and fixed effects of line, on-test group, sex, and interactions of generation by line with each of the following covariates: onwt - 40, age at on-test - 90, offwt - 115, metamidwt, adga, and offbfa.

### Genotyping

DNA was isolated from tail tissue using the Qiagen (Valencia, CA, USA) DNeasy blood & tissue kit. Genotyping was completed with the Illumina (San Diego, CA, USA) PorcineSNP60 BeadChip by GeneSeek, Inc. (Lincoln, NE, USA) with approved standard techniques outlined by the manufacturer. A total of 50,953 SNPs that met quality control criteria (>80% call rate, >40% GC score and Hardy Weinberg equilibrium P value>0.0001) were used.

### Population stratification

Population stratification was analyzed by identity-by-state (IBS) and multi dimensional scaling (MDS) clustering methods available in the PLINK software. Diagnostic tools to understand the stratification included Q-Q plots based on the associated P values for individual SNPs and haplotypes from the PLINK software.

### Allele frequency differences

The allele frequency differences method used animals from generation 8 of the low (n = 78) and high (n = 79) RFI lines, as allele frequencies are expected to have been changing over generations as the divergent selection continued. Allele frequencies for each line were calculated for each SNP, and then used to compute differences between lines. Allele frequency differences were categorized into five groups based on minor allele frequencies (MAF) (<0.1, 0.1–0.19, 0.2–0.29, 0.3–0.39, 0.4–0.5) of the SNP in generation zero. This step was to control for the effect of genetic drift, which is expected to be greater for SNP with low MAF. Later, the mean and standard deviations (SD) of allele frequencies were calculated for the SNP in each group, and were used to obtain Z scores for allele frequency differences in each group. The P-values corresponding to each Z score were log transformed and plotted using R software [21]. Multiple testing was corrected for by using the Benjamini-Hochberg false discovery rate [22], as explained by Agilent Technologies Inc. technical support documentation (http://www.chem.agilent.com/cag/bsp/sig/downloads/pdf/mtc.pdf, Accessed 2012 October 20^th^).

### Whole genome association studies

Two approaches were followed for association analyses. Primarily, a Bayesian approach based on genomic selection called Bayes B [23], as implemented in GenSel software [24], was used to obtain the variance explained by SNPs in every genomic window of one mega base (Mb). The statistical model used for the Bayes B approach was:
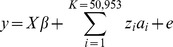
where 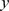 is a vector of phenotypes; 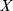 is an incidence matrix of fixed effects (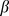); 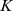 is the total number of SNPs; 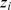 is a vector of genotypes of a fitted marker 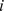, coded -10/0/10; 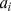 is a random substitution effect of fitted marker 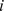 with its own variance 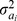 and a priori zero effect with probability π or a non-zero effect with probability 1-π , as explained by [25]; and *e* is the vector of random residuals assumed to be normally distributed. The fixed effects used in this model were sex and pen by group. Fixed covariates were age when animals were put on-test, with a different slope for each combination of selection line and parity. As explained in detail in our earlier papers [11], [12], [13] for reproductive and structural traits, the current analyses for Bayes B utilized the same prior probability pi of 0.995, so as to fit 250–300 markers per iteration of the Markov chain in a mixture model for the estimation of individual SNP effects. A total of 51,050 iterations of a Markov chain were run for the analyses, with 1000 iterations burn-in and 50 iterations as an output frequency. The current analyses used a Bayes B rather than a Bayes C approach because of its better performance over Bayes C for QTL mapping with 1 Mb genomic windows in a comparative study of different Bayesian methods for QTL mapping [26].

The pig genome includes 2,815 non-overlapping 1 Mb windows based on the 50,953 genotyped markers. Therefore, the expected percent of genetic variance accounted for by one window was 100%/2815, which was nearly 0.04%. Hence, 1 Mb windows that explained at least 0.2% of genetic variance, which is 5 times greater than expected (0.04%*5 = 0.2%), were considered to contain putative QTL. The markers from unassigned contigs and those completely unmapped to any contig or the genome were considered as unmapped markers and were not included in the results. The SNP in QTL regions were considered for further haplotype analyses to determine their association with the studied traits.

Positional candidate genes were searched in the putative QTL regions and neighboring upstream and downstream 1 Mb regions based on *Sus scrofa* genome build 10.2 (http://www.ensembl.org/Sus_scrofa/Info/Index, Accessed 2012 September 20^th^). Protein sequences of those genes, which were not annotated with a specific name in these regions in *Sus scrofa* genome build 10.2, were used in NCBI-BLAST to identify their names based on homologous sequences from other species with an E value <1e09. The gene functions were examined by using a literature search. Previously reported QTL in these regions were obtained from the GBrowse option in Animal QTLdb (http://www.animalgenome.org/cgi-bin/gbrowse/pig/, Accessed 2012 October 10^th^) on the basis of *Sus scrofa* genome build 10.2.

Single SNP association analyses were performed using PLINK software [27]. Prior to PLINK analyses, phenotypes were adjusted for fixed factors of sex and pen by group and a covariate of age when animals were put on-test with a different slope for each combination of selection line and parity by using SAS 9.2 software (Version 9.2, SAS Institute Inc, Cary, NC, USA). The adjusted phenotypes were used in the association analyses with the PLINK basic “–assoc” command. To control for population structure and to perform multiple testing, genomic control (GC) followed by FDR was implemented on the empirical P values using the “–gc –adjust” command in PLINK software [28]. As no SNP was significantly associated with the studied traits after GC and FDR, except for LMA, associations were considered significant based on GC corrected P values at a threshold of 0.01, as earlier reported by [28].

### Haplotype Association Analyses (HAA)

For each trait, SNP from 1 Mb genomic window regions that explained at least 0.2% of genetic variance in the Bayes B approach were selected for construction of linkage disequilibrium (LD) blocks. The LD blocks were constructed using the method of confidence intervals and default parameters of the Haploview v4.1 software [29]. Haplotypes for each LD block were determined by PHASE software version 2.1 [30], [31] for all animals. Haplotypes with a frequency greater than 5% in the population were considered for further association analyses with phenotypes adjusted for the same fixed factors and covariates as used for BayesB, but by using PLINK software. Similar to the top single SNP associations, the top haplotypes were considered based on GC-corrected P value at a threshold of 0.05. These analyses consider only 1 Mb genomic regions that were pre-selected for association, thus the results need to be considered cautiously.

Raw data will be shared upon request.

## Results and Discussion

### Population stratification

**Figure 2:**
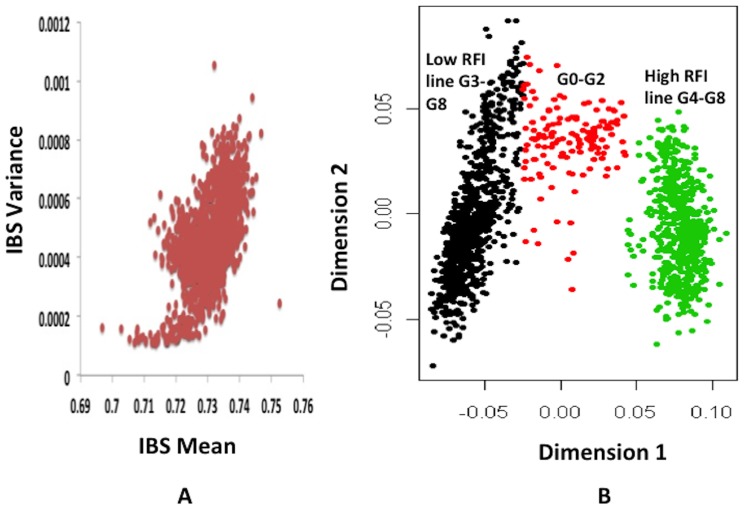
Clustering of the residual feed intake (RFI) population by the PLINK software. **Figure A indicates identical-by-state (IBS) clustering. The X and Y axes represent IBS mean and IBS variance. Figure B shows multi-dimensional clustering.** The X and Y-axes indicate dimensions 1 and 2, respectively. The black color cluster includes animals from generations 3–8 of the Low RFI line, the green cluster includes animals from generations 4–8 of the High RFI line, and the red cluster includes animals from generations 1 and 2 of the Low RFI line and generation 0, which is the founder population from which RFI selection lines originated.

Population stratification with IBS clustering showed that all animals belonged to one cluster (Figure 2A). However, MDS clustering identified three clusters, separating animals from generations G0–G2 and G3–G8 of the low RFI line and G4–G8 of the high RFI line (Figure 2B). The IBS clustering is based on a similarity matrix depending on the identical-by-state nature of the genotypes among the individuals. There may be high IBS similarity even between unrelated individuals just by chance. But MDS is based on a dissimilarity matrix (1- similarity or IBS matrix) in multiple dimensions. Hence, MDS clustering is more preferable than the IBS clustering [27]. For the single marker and haplotype association analyses, stratification was corrected by genomic control, using the PLINK software, as illustrated by Q-Q plots in Figures S1 and S2. Stratification is implicitly accounted for by fitting all markers simultaneously in the genomic selection analyses based Bayesian approaches in the GenSel software [32].

### Allele frequency differences

**Figure 3:**
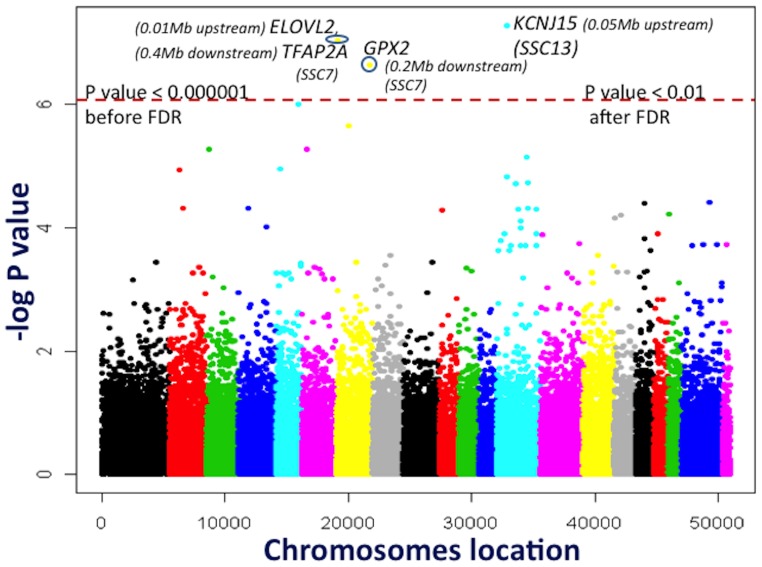
Allele frequency differences between the Low and High residual feed intake lines at generation 8. The X axis indicates in different colors from left to right, SNP locations from chromosomes 1 to X, unassigned contigs, Y, and completely unmapped SNPs, using *Sus scrofa* genome build10.2. The Y axis represents the minus log of the P-value for the allele frequency difference between the two lines for each SNP,. The dashed line shows the P-value threshold. SSC: Pig chromosome; FDR: False discovery rate; *KCNJ15:* Potassium inwardly rectifying channel, subfamily J, member 15; *ELOVL2*: Elongation of very long chain fatty acids 2; *TFAP2A*: Transcription factor AP-2 (Activating enhancer binding protein 2)—alpha; *GPX2*: Glutathione peroxidase 2.

**Figure 4:**
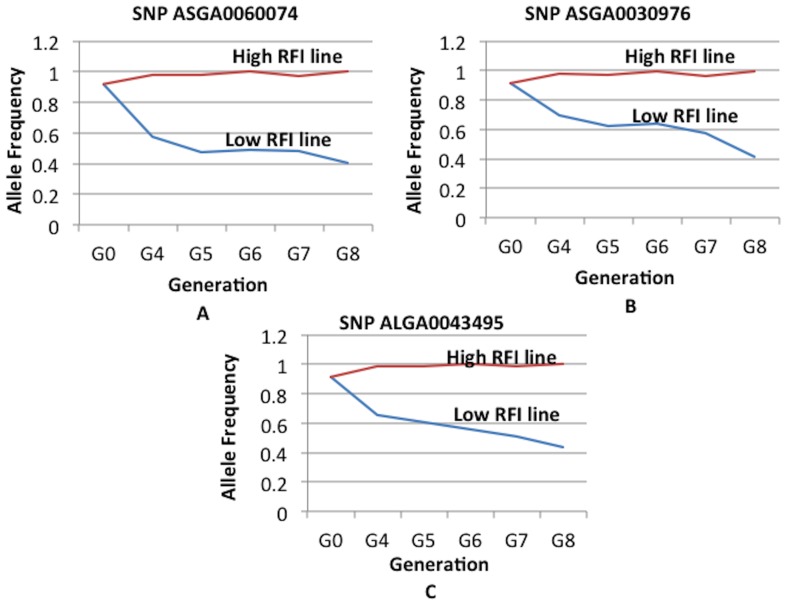
Allele frequency differences between the residual feed intake selection lines for the significant SNPs in each generation. Parts A, B and C show the allele frequency differences for the SNPs near to the *KCNJ15* gene on SSC13 (ASGA0060074), near the *ELOVL2* and *TFAP2A* gene on SSC7 (ASGA0030976), and near the GPX2 gene on SSC7 (ALGA0043495), respectively. *KCNJ15:* potassium inwardly rectifying channel, subfamily J, member 15; *ELOVL2*: elongation of very long chain fatty acids 2; *TFAP2A*: transcription factor AP-2 (activating enhancer binding protein 2)—alpha; *GPX2*: glutathione peroxidase 2. The X axes represent generations and Y-axes show allele frequencies.

Some SNP on SSC13 (ASGA0060074) and SSC7 (ASGA0030976, ALGA0043495) showed very significant (P<0.000001, FDR P value<0.01) differences in allele frequencies between the high and low RFI lines at generation 8 (Figure 3 and Table 1), the last available generation. Interestingly, the *KCNJ15* gene (potassium inwardly rectifying channel, subfamily J, member 15) is located within 0.05 Mb upstream of SNP ASGA0060074 (T/C) on SSC 13, which had the most significant difference in allele frequencies between the lines (0.40 in Low RFI line; 1.0 in High RFI line for the allele “T”) (Figures 3 and 4). The *KCNJ15* gene encodes one of the potassium inward rectifying channels and is a type-2 diabetes risk gene and its up-regulation causes down regulation of glucose induced insulin release in mice and humans [33]. Similarly, the *ELOVL2* gene (elongation of very long chain fatty acids 2) is located within 0.01 Mb upstream from SNP ASGA0030976 on SSC7 and is involved in enhancing the synthesis of triglycerides and deposition of lipid droplets in adipocytes [34]. The adipocytes usually produce leptin due to fat deposition [35]. It is well known that circulating insulin and leptin reduce food intake through their central actions in the brain [36], [37]. This may explain why the RFI selection lines might differ in alleles related to insulin and leptin gene regulation. Additionally, the genes *TFAP2A* (transcription factor AP-2 (activating enhancer binding protein 2) - alpha), a master regulator of other transcription factors in mouse liver [38], and *GPX2* (glutathione peroxidase 2), an isozyme responsible for the majority of the glutathione dependent hydrogen peroxide reducing activity in the gastrointestinal tract (http://www.ncbi.nlm.nih.gov/gene/2877, Accessed 2012 June 10^th^), are located within 0.4 Mb downstream of SNP ASGA0030976 and within 0.2 Mb downstream of SNP ALGA0043495, respectively, on SSC7. This indicates that the RFI selection lines showed significant differences in allele frequencies near genes involved in metabolism in the liver and the gastrointestinal tract. Further functional studies with different tissues from RFI selection lines need to be conducted to confirm the role of these genes in the RFI phenotype.

**Table 1:** SNPs with significant (P value<0.0001 and FDR P value<0.05) allele frequency differences in generation 8 between Low and High RFI selection lines.

SSC	SNP	Location of SNP	P value before FDR	−log P value	P Value after FDR	Genes within 0.5 Mb upstream of the SNP	Genes within 0.5 Mb downstream of the SNP
13	ASGA0060074	211966700	5.25E-08	7.27	0.002	*SNORA72*	*KCNJ15*
7	ASGA0030976	7981110	9.24E-08	7.03	0.002	*TFAP2A, GCNT2, MAK, C6ORF52, TMEMB14*	*ELOVL2, NEDD9,*
7	ALGA0043495	95603020	2.33E-07	6.63	0.003	*GPX2, MAX, FNTB, CHURC1, SPTB, PLEKHG3, RAB15*	*FUT8*
5	MARC0013717	63387978	9.94E-07	6.00	0.012	*ETV6, SNORA22, 5srRNA*	*TCTP, LRP6, BCL-G, Novel transcript*
7	H3GA0019660	5155195	2.24E-06	5.64	0.022	*SSR1, RIOK1, DSP, SNRNP48*	*BMP6, EEF1E1, MUTED, LOC100515671*
6	ALGA0122972	8687182	5.30E-06	5.27	0.038	*No annotated genes*	*5s-rRNA, CLEC-34A, WWOX*
3	ALGA0114825	79728805	5.30E-06	5.27	0.04	*MEIS1*	*No annotated genes*
13	MARC0069512	123984019	7.21E-06	5.14	0.04	*No annotated genes*	*TBL1XR1, LOC100523210*

### WGAS and HAA for RFI

**Figure 5:**
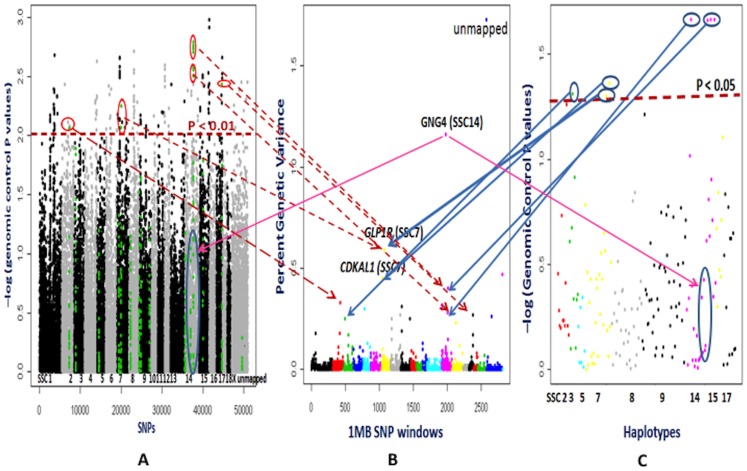
Whole genome association studies for residual feed intake (RFI). **Part A** depicts association analyses performed by the PLINK software for each SNP. The X axis shows SNPs across chromosomes 1 to X, unassigned contigs, Y and completely unmapped SNP. The Y axis represents the negative logarithm of the P-values corrected for genomic control. Each spot is a SNP. The green color SNPs are those located in 1 Mb window regions that explain more than 0.2% of genetic variance in part B. **Part B** illustrates results from the Bayes B model averaging approach used in the GenSel software. Different colors on the X axis indicate genome wide 1 Mb SNP windows from chromosome 1 to X, unassigned contigs, Y and completely unmapped SNP. The markers from completely unmapped and unassigned contigs were not included in the cumulative genetic variance. The Y axis represents percent genetic variance explained by each 1 Mb window. **Part C** shows association analyses with the PLINK software based on haplotypes, which were derived for 1 Mb windows that explained a higher than 0.2% of genetic variance in part B. The X axis shows chromosomal positions of the haplotypes. The Y axis shows the negative logarithm of the P-values corrected by genomic control. The arrows in parts A, B and C show the similarities in the significant locations. The 1 Mb windows that explained a higher than 0.2% percent of genetic variance in the Bayesian analyses and/or were significant in the PLINK analyses were considered to be important putative QTL for RFI. *GNG4*: guanine nucleotide binding protein 4; *GLP1R*: glucagon-like peptide 1 receptor; *CDKAL1:* cyclin-dependent kinase 5 regulatory subunit associated protein 1-like 1.

WGAS were carried out with a larger population comprising both the lines and all genotyped individuals from generation 0 to generation 8. The Bayes B approach in the GenSel software revealed that the genomic heritability or proportion of phenotypic variance captured by genome wide markers was 0.52 for RFI, which is a little higher than the genomic heritabilities obtained for ADFI, ADG, BF and LMA and indicates that RFI is moderate to highly heritable (Table S1). For the Bayes B approach, a 1 Mb SNP window (H3GA0040291-MARC0009335) at 59 Mb on SSC14 explained more than 1% of the genetic variance in RFI with a posterior probability of association 0.68 (p>0; PPA) or a false positive probability (1-p>0) of 0.32 (Figure 5 and Tables 2 and S2). The *GNG4* (guanine nucleotide binding protein 4) gene in this region is a trimeric G protein with alpha, beta and gamma subunits. In its active state, the Gbetagamma subunit of *GNG4* can activate potassium inward rectifying channels (http://www.genecards.org/cgi-bin/carddisp.pl?gene=GNG4, Accessed 2012 June 20^th^) in conjunction with M1 (Muscarinic 1 acetylcholine) receptors. The importance of potassium inward rectifying channels was emphasized in the allele frequency differences between high and low RFI lines in generation 8 for *KCNJ15* and again here with results involving *GNG4* for RFI. The second most significant 1 Mb SNP window (ALGA0040519-ASGA0032851), explaining 0.59% of the genetic variance for RFI with a PPA of 0.48, is on SSC7 at 39 Mb. This window also includes two potassium channels (*KCNK5, KCNK17*), one of which (*KCNK17* also known as *TALK2*) is highly expressed in pancreas in humans [39]. Interestingly, the gene *GLP1R* (glucagon-like peptide 1 receptor) in this window on SSC7 increases synthesis and release of insulin from the pancreas by activating the adenylyl cyclase pathway [40] and has also been reported to be involved in appetite control. The third most significant 1 Mb SNP window (ALGA0038863-DRGA0007204) was also located on SSC7 but at 16 Mb. This window explained 0.45% of the genetic variance, with a PPA of 0.41. This window also contains an insulin release-regulating gene called *CDKAL1* (cyclin-dependent kinase 5 regulatory subunit associated protein 1-like 1), which is involved in the first phase insulin release through provision of ATP and potassium-ATP channel responsiveness [41]. Based on the allele frequency differences and Bayesian analyses, the genomic regions containing genes involved in insulin release could partly explain the biology of differences in RFI in the RFI selection lines, although these regions have only modest PPA (Figure 5, and Tables 2 and S2). Significant (P<0.05) plasma insulin differences were also observed between low and high RFI selection lines in another pig population [42].

**Table 2:** Important candidate QTL regions associated with the residual feed intake (RFI) by 1 Mb SNP windows.

SSC	Location (start-end) in Mb@	1 Mb SNP window	Percent genetic variance explained	PPA* (P>0)	Genes within the SNP window^$^	Previously reported important QTL at the SNP window
14	59.00–59.98	H3GA0040291-MARC0009335	1.16	0.682	*GPR137B, LYST, GNG4, B3GALNT, TBCE*	Daily feed intake and body weight
7	39.01–39.98	ALGA0040519-ASGA0032851	0.59	0.480	*BTBD9, GLO1, DNAH8, GLP1R, KCNK5, KCNK17*	Average daily gain and body weight
7	16.06–16.97	ALGA0038863-DRGA0007204	0.45	0.412	*ID4, MBOAT1, E2F3, CDKAL1*	Average daily gain and body weight
14	90.03–90.96	ASGA0064826-ALGA0079379	0.40	0.336	*5S - rRNA*	Average daily gain and body weight

@ The 1 Mb windows are presented in descending order based on the percent genetic variance explained greater than 0.4%.

* Posterior probability that the SNPs in 1 Mb window could explain the genetic variance greater than zero (PPA: Posterior probability of association).

The PLINK analyses supported the association (P<0.01 after GC) of individual SNP in the 1 Mb SNP window (ALGA0040519-ASGA0032851) at 39 Mb on SSC7 that contains the *GLP1R* gene (Figure 5 and Table S2). Similarly, HAA also revealed a significant (P<0.05 after GC) association of the -GTATATTT- haplotype in this 1 Mb SNP window, and also of the -GGGCGTAA- haplotype in the 1 Mb SNP window that contains the *CDKAL1* gene on SSC7. Both PLINK and HAA analyses showed significant associations for SNP (P<0.01 after GC) and haplotypes (P<0.05 after GC) in the 1 Mb SNP windows on SSC14 that explained 0.4% and 0.32% of genetic variance in the Bayes B approach, with a PPA of 0.28. However, the SNPs and haplotypes in the 1 Mb SNP window that contains the *GNG4* gene on SSC14 did not show significant associations in the PLINK analyses (Figure 5 and Table S2). No SNP showed a significant association with RFI after genomic control followed by FDR in the PLINK analyses. Hence, GC corrected P values were considered for an association threshold as a previous report had followed the similar consideration [28]. This observation suggests that analyzing windows of neighboring SNPs using genomic selection based Bayesian methods may be more useful than frequentist approaches for understanding the biology of traits using these types of data sets.

### WGAS and HAA for ADFI

**Figure 6:**
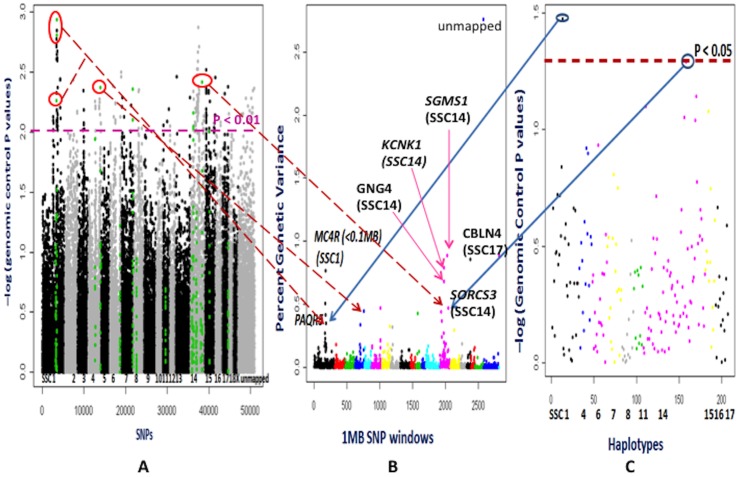
Whole genome association studies for average daily feed intake (ADFI). **Part A** depicts association analyses performed by the PLINK software for each SNPs. The X axis shows SNPs across chromosomes 1 to X, unassigned contigs, Y and completely unmapped SNP. The Y axis represents the negative logarithm of the P values corrected for genomic control. Each spot is a SNP. The green color SNPs are those located in 1 Mb window regions that explain more than 0.2% of genetic variance in part B. **Part B** illustrates results from the Bayes B model averaging approach used in the GenSel software. Different colors on the X axis indicate genome wide 1 Mb SNP windows from chromosome 1 to X, unassigned contigs, Y and completely unmapped SNP. The markers from completely unmapped and unassigned contigs were not included in the cumulative genetic variance. The Y axis represents percent genetic variance explained by each 1 Mb window. **Part C** shows association analyses with the PLINK software based on haplotypes, which were derived for 1 Mb windows that explained a higher than 0.2% of genetic variance in part B. The X axis depicts chromosomal positions of haplotypes. The Y axis shows the negative logarithm of the P values corrected by genomic control. The arrows in parts A, B and C show the similarities in significant locations of the associated SNPs, SNP windows and their haplotypes. The 1 Mb windows that explained higher than 0.2% percent genetic variance in GenSel analyses and/or were significant in the PLINK analyses were considered to be important putative QTL for ADFI. *SGMS1*: spingomyelin synthase 1; *CBLN4*: cerebellin 4; *KCNK1*: potassium channel, subfamily K, member 1; *MC4R*: melanocortin 4 receptor; *PAQR5*: progestin and adipoQ receptor family member V; *GNG4*: guanine nucleotide binding protein 4; *SORCS3*: sortilin-related vps10 domain containing receptor 3.

In the Bayes B approach, several 1 Mb SNP windows, including at 107 Mb (ASGA0065520-ALGA0080315) on SSC 14 and at 63 Mb (ALGA0096110-M1GA0022378) on SSC17, explained more than 0.8% of the genetic variance in ADFI, with a PPA greater than 0.5 (Figure 6 and Table S3). The genes in these regions, specifically *SGMS1* (spingomyelin synthase 1) on SSC14 and *CBLN4* (cerebellin 4) on SSC 17, are important for brain functions (RefSeq [43]) and for insulin secretion from pancreatic cells [44], [45]. Specially, *sgsm1* null mice showed impaired insulin secretion due to increased reactive oxygen species and mitochondrial dysfunction [44]. The genes *PRKG1* (cGMP-dependent protein kinase 1, alpha isozyme) and *PTEN* (tumor-suppressor phosphatase and tensin homologue) are located within 1 Mb upstream and downstream, respectively, to the genomic window at 107 Mb on SSC 14. In beef cattle, an intronic SNP (rs29013727) in the *PRKG1* gene was significantly (P<0.05) associated with dry matter intake (DMI) by whole genome single SNP association analyses [46]. Mutations in the *PTEN* gene enhanced insulin sensitivity by modulating the PI3K-AKT pathway [47]. The *BMP7* (bone morphogenic protein 7) and *MC3R* (melanocortin-3 receptor) genes are located within 1 Mb downstream of the genomic window (ALGA0096110-M1GA0022378) on SSC17 at 63 Mb. The BMP7 protein reduces food intake and increases energy expenditure through its leptin-independent mechanism such as central mTOR-p70S6 kinase pathway [48]. The MC3R protein is involved in regulating feeding behavior and metabolism by anticipating nutrient intake through the extracellular regulating kinase (ERK) pathway in the dorsomedial hypothalamus (DMH) and is thus helpful in adaptation during restricted feeding [49]. These observations reinforce the importance of also studying genes located in neighboring regions of the most highly associated genomic windows.

The third most significant 1 Mb SNP window (MARC0044077-ALGA0077929) on SSC14 at 61 Mb, explained 0.79% of the genetic variance, with a PPA of 0.469, and contains another potassium channel *KCNK1* (potassium channel, subfamily K, member 1) gene. Interestingly, a 1 Mb SNP window region (ALGA0006599 - INRA0004954) close (<0.1 Mb upstream) to *MC4R* (melanocortin 4 receptor) at 177 MbMb on SSC1) explained 0.77% of the genetic variance, with a PPA of 0.30. The current Illumina PorcineSNP60 BeadChip does not have a SNP in the *MC4R* gene. Therefore, this nearby window might not capture the full effect of *MC4R* on ADFI, considering that *MC4R* is well known to be associated with feed intake in pigs [50], [51] through its involvement in leptin [52] and insulin actions [53]. The 1 Mb SNP window containing *GNG4* on SSC14, which was also associated with RFI, explained 0.68% of the genetic variance for ADFI and had a PPA of 0.38 (Figure 6 and Table S3). All these mapped genomic regions, except those on SSC17, contained previously reported QTL for feed intake (Table S3). Moreover, the genes in these regions that were identified using the Bayesian approach reinforce hypothesis of the regulation of insulin release and sensitivity for feed intake. The PLINK and HAA analyses showed significant association for individual SNP (P<0.01 after GC) and haplotypes (P<0.05 after GC) in the 1 Mb window at 185 Mb on SSC1 (ALGA0006854 - H3GA0003303 at 185 Mb). This window contains the *PAQR5* gene (progestin and adipoQ receptor family member V) and it explained 0.35% of genetic variance in the Bayesian analysis, with a PPA of 0.265. Although the PLINK and HAA analyses showed no significant associations after accounting for multiple testing, this modest association of *PAQR5* containing genomic window with ADFI can be cautiously considered to be important due to the physiological function of *PAQR5*. This non-genomic progesterone membrane receptor facilitates the actions of progesterone on glucose homeostasis through the secretion of incretin (hormones enhancing insulin secretion) from enteroendocrine cells [54].

### WGAS and HAA for ADG

**Figure 7:**
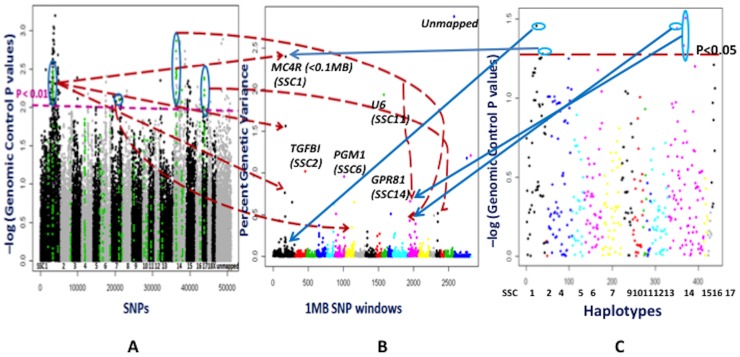
Whole genome association analyses for average daily gain (ADG). **Part A** depicts association analyses performed by the PLINK software for each SNP. The X axis shows SNPs across chromosomes SSC1 to X, unassigned contigs, Y and completely unmapped SNP. The Y axis contains the negative logarithm of the P values adjusted for genomic control. Each spot is a SNP. The green color SNPs are those located in 1 Mb window regions that explain more than 0.2% of genetic variance in part B. **Part B** illustrates results from the Bayes B model averaging approach used in the Gensel software. Different colors on the X axis indicate genome wide 1MB SNP windows from chromosome 1 to X, unassigned contigs and completely unmapped SNP. The markers from completely unmapped and unassigned contigs were not included in the cumulative genetic variance. The Y axis represents percent genetic variance explained by each 1 Mb window. **Part C** shows association analyses with the PLINK software based on haplotypes, which were derived for 1 Mb windows that explained a higher than 0.2% of genetic variance in part B. The X axis has haplotypes on specific chromosomes. The Y axis shows the negative logarithm of the P values corrected by genomic control. The arrows in parts A, B and C show the similarities in significant locations of the associated SNPs, SNP windows and their haplotypes. The 1 Mb windows that explained higher than 0.2% percent genetic variance in Gensel analyses and/or were significant in the PLINK analyses were considered to be important putative QTL for ADG. *MC4R*: melanocortin 4 receptor; *TGFBI*: transforming growth factor beta induced protein ig-h3; *PGM1*: phosphoglucomutase 1; *GPR81*: G-protein coupled receptor 81; U6: spliceosomal RNA.

The most significant 1 Mb window (ALGA0006599-INRA0004954) in the Bayesian approach was on SSC1 at 177 Mb (Figure 7 and Table S4), which explained 2.4% of genetic variance of ADG, with a PPA of 0.427. The *MC4R* gene is near (<0.1 Mb downstream) this genomic window, as described above for ADFI. In pigs, SNPs in *MC4R* have not only been shown to be associated with ADFI but also with ADG in many studies [51], [55], [56], [57]. This significant association was supported by the single SNP analyses with PLINK (P<0.01 after GC) and by HAA (P<0.05 after GC) in this window (Figure 7 and Table S4). Neighboring 1 Mb SNP windows at 176 Mb (INRA0004873 - ASGA0004980) and at 167 Mb (MARC0096493 - MARC0054709) explained 1.56% and 0.76% of genetic variance in the Bayesian approach, with PPA 0.30 and 0.16, respectively. These results suggest that the QTL spanning these genomic windows on SSC1 can quite confidently be associated with ADG in the RFI selection lines. Furthermore, the 1 Mb SNP windows on SSC11 (H3GA0031714-ALGA0061627 at 28 Mb), on SSC2 (ASGA0098016-ASGA0085390 at 143 Mb), on SSC6 (DIAS0000949-DRGA0006954 at 137 Mb), and on SSC14 (INRA0043392-ALGA0076686 at 31 Mb), respectively, explained 1.94% (PPA = 0.664), 1.02% (PPA = 0.532), 0.96% (PPA = 0.443) and 0.66% (PPA = 0.341) of genetic variance (Figure 7 and Table S4). The window on SSC11 does not have any annotated protein coding genes in the current pig genome build. However, the *OLFM4* (olfactomedin 4) gene is within 1 Mb upstream to the genomic window at 28 Mb on SSC11. The *OLFM4* gene encodes a secreted glycoprotein that helps cell adhesion through lectins and cadherins on the cell surface, and some observations have linked it to innate immunity and gut microflora [58]. A strong association of a SNP close (500 kb) to the *OLFM4* gene with childhood obesity in a genome-wide meta-analysis supported its possible role in the relationship between gut microbiome and obesity risk [59]. Among the genes in the window (ASGA0098016-ASGA0085390) at 143 Mb on SSC2, *TGFBI* (transforming growth factor beta induced protein ig-h3) is an extracellular matrix protein, which has a role in myofibril bundling and muscle fiber growth [60]. A key glucose metabolism gene called *PGM1* (phosphoglucomutase 1) is one of the genes in the window (DIAS0000949-DRGA0006954) at 137 Mb on SSC6. Differential expression of *PGM1* is associated with the glycolytic potential of muscle and its energy dependence on either fat or glucose, which is a determinant of lean meat production [61]. Another energy metabolism gene, *GPR81* (G-protein coupled receptor 81), whose expression is restricted to only adipose tissues, is located on SSC14 (INRA0043392-ALGA0076686) at 31 Mb. GPR81 facilitates the anti-lipolytic activity of lactate, a product of glycolysis during exercise and oxygen deficit [62]. Individual SNPs and haplotypes in this window on SSC14 also showed significant associations based on the GC corrected P values (Figure 7 and Table S4). Taken together, the genomic regions that include genes associated with energy homeostasis genes (e.g., *MC4R* (SSC1), *OLFM4* (SSC11), *PGM1* (SSC6) and *GPR81* (SSC14)) and muscle growth (e.g., *TGFB1* (SSC2)) might be associated with ADG in the RFI population.

### WGAS and HAA for BF

**Figure 8:**
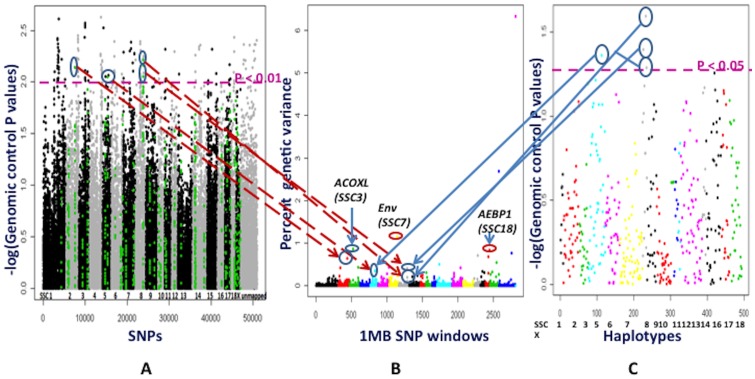
Whole genome association studies for back fat (BF). **Part A** depicts association analyses performed by the PLINK software for each SNP. The X axis shows SNPs across chromosomes SSC1 to X, unassigned cintigs, Y and completely unmapped SNP. The Y axis contains the negative logarithm of the P values adjusted for genomic control. Each spot is a SNP. The green color SNPs are those located in 1 Mb window regions that explain more than 0.2% of genetic variance in part B. **Part B** illustrates results from the Bayes B model averaging approach used in the Gensel software. Different colors on the X axis indicate genome wide 1 Mb SNP windows from chromosome 1 to X, unassigned contigs and completely unmapped SNP. The markers from completely unmapped and unassigned contigs were not included in the cumulative genetic variance. The Y axis represents percent genetic variance explained by each 1 Mb window. **Part C** shows association analyses with the PLINK software based on haplotypes, which were derived for 1 Mb windows that explained a higher than 0.2% of genetic variance in part B. The X axis has haplotypes on specific chromosomes. The Y axis shows the negative logarithm of the P values corrected by genomic control. The arrows in parts A, B and C show the similarities in significant locations of the associated SNPs, SNP windows and their haplotypes. The 1 Mb windows that explained higher than 0.2% percent genetic variance in Gensel analyses and/or were significant in the PLINK analyses were considered to be important putative QTL for BF. *ACOXL*: acyl-coenzyme A oxidase-like; *AEBP1*: adipocyte enhancer binding protein 1; *Env*: envelope protein.

The Bayesian approach identified 1 Mb windows on SSC7 (ALGA0044374-DRGA0008090) at 112 Mb, on SSC3 (MARC0085867-ALGA0018683) at 47 Mb, and on SSC18 (ALGA0119800-ALGA0123577) at 55 Mb to explain higher genetic variance (>0.8% with PPA>0.50) than other 1 Mb windows (Figure 8 and Table S5). Windows contributed by unmapped markers were ignored as they do not represent true consecutive SNP windows. No fat related genes have been annotated in the 1 Mb window (ALGA0044374-DRGA0008090) on SSC7 at 112 Mb. However, 1 Mb windows on SSC3 at 47 Mb (MARC0085867-ALGA0018683) and on SSC18 at 55 Mb (ALGA0119800-ALGA0123577) contain important fat metabolism genes which increases the likelihood that these regions affect back fat deposition. The ACOXL (acyl-coenzyme A oxidase-like) in the 47 Mb window on SSC3 has acyl-CoA dehydrogenase activity and catalyzes an important step in the ß-oxidation pathway for the oxidation of long chain fatty acids (http://www.genecards.org/cgi-bin/carddisp.pl?gene=ACOXL, Accessed on 2012 June 10^th^ ). Additionally, the *GPAT2* gene (glycerol-3-phosphate acyltransferase 2, mitochondrial), an isoform that catalyzes the first step in triglyceride synthesis [63] is located within 1 Mb downstream of this window (MARC0085867-ALGA0018683) on SSC3. Similarly, the gene *AEBP1* gene (adipocyte enhancer binding protein 1) is located in the 1 Mb window (ALGA0119800-ALGA0123577) at 55 Mb on SSC18 and is a transcriptional repressor that regulates the expression of fatty acid binding protein 4 (FABP4) by binding to a regulatory element called adipocyte enhancer 1 (AE1) in the proximal promoter *FABP4* gene (http://www.ncbi.nlm.nih.gov/gene/165#reference-sequences, Accessed on 2012 June 10^th^). Higher expression of FABP4 in adipose tissue has been found to be associated with leanness in humans [64] and higher marbling in pigs [65]. Although these associated genomic regions from the Bayesian analyses were not supported by single SNP analyses with the PLINK and HAA approaches after genomic control (Figure 8), the physiological functions of the genes and PPA greater than 0.5 support the consideration of these regions as QTL for BF in the RFI selection lines (Figure 8).

### WGAS and HAA for LMA

**Figure 9:**
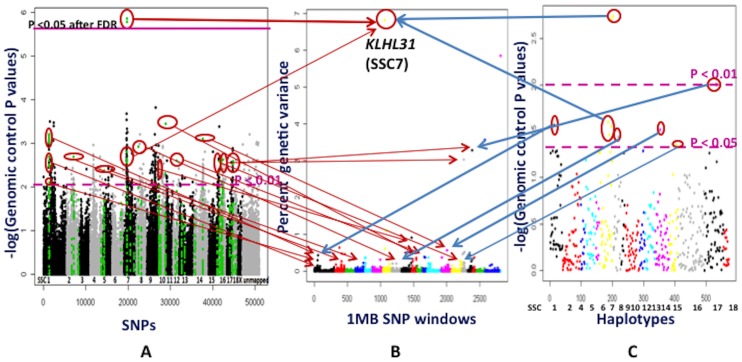
Whole genome association studies for loin muscle area (LMA). **Part A** depicts association analyses performed by the PLINK software for each SNP. The X axis shows SNPs across chromosomes SSC1 to X, unassigned contigs, Y and unmapped SNP. The Y axis contains the negative logarithm of the P values adjusted for genomic control. Each spot is a SNP. The green color SNPs are those located in 1 Mb window regions that explain more than 0.2% of genetic variance in part B. **Part B** illustrates results from the Bayes B model averaging approach used in the Gensel software. Different colors on the X axis indicate genome wide 1 Mb SNP windows from chromosome 1 to X, unassigned contigs, Y and completely unmapped SNP. The markers from completely unmapped and unassigned contigs were not included in the cumulative genetic variance. The Y axis represents percent genetic variance explained by each 1 Mb window. **Part C** shows association analyses with the PLINK software based on haplotypes, which were selected from the 1 Mb windows that explained a higher than 0.2% of genetic variance in part B. The X axis has haplotypes on specific chromosomes. The Y axis shows the negative logarithm of the P values corrected by genomic control. The arrows in parts A, B and C show the similarities in significant locations of the associated SNPs, SNP windows and their haplotypes. The 1 Mb windows that explained higher than 0.2% percent genetic variance in Gensel analyses and/or were very highly significant even after genomic control followed by FDR (P value<0.05) in the PLINK analyses were considered to be important QTL for LMA. *KLHL31*: kelch like 31.

A 1 Mb window (ALGA0039868-ASGA0032245) at 31 Mb on SSC7 that contains the *KLHL31* (kelch like 31) gene explained 6.82% of the genetic variance for LMA, with a PPA of 0.93 (Figure 9 and Table S6) based on the Bayesian approach. Two SNPs (H3GA0020592 and MARC0010879) in this region were very significantly (P<0.00001 after GC) associated with LMA in the PLINK analyses, even after GC followed by FDR (P<0.05) (Figures S1 and 9). Haplotypes in this region were also very significantly (P<0.01 after GC) associated with LMA (Figures S2 and 9). Based on these observations, this genomic region can be considered as a new QTL for LMA, as no previous QTL were reported in this region for LMA (Table S6). The *KLHL31* gene is the best candidate gene in this region for further fine mapping based on its role in skeletal myogenesis [66]. The closest region that was previously identified to be associated with LMA on SSC7 contains the major histocompatibility complex [67] and is located 2 Mb upstream of the region identified in this study.

### General comparisons among different analyses

In general, the results from allele frequency difference method showed very significant differences between the high and low RFI lines at certain SNPs (P<0.000001, FDR P value<0.01) (Figures 3 and 4). These significant SNPs (ASGA0060074 on SSC13, ASGA0030976 and ALGA0043495 on SSC7) did not show significant associations in other analyses, as allele frequency differences used only generation 8 animals. The empirical P values in the single SNP and haplotype analyses by the PLINK software were inflated from the expected significance level due to population stratification (Figures S1 and S2). Genomic control correction of population stratification followed by multiple corrections with FDR resulted in no significant associations for single SNP and haplotypes for all traits except for LMA. Hence, the associations of genomic regions detected using the Bayesian approach were emphasized as the Bayesian approaches not only use the information that could be obtained from the data but also they can borrow the information (priors) from other similar studies [68], and these approaches can select a certain proportion of the markers from whole genome markers in an iterative manner. Additionally, the BayesB approach considers different genetic variation for the different markers, which is closer to the biological situation of quantitative traits [69]. The results from genomic control corrected P values for single SNPs and haplotypes within these genomic regions were considered as supportive evidence. Only the single SNPs within the associated genomic regions from the Bayesian approach were highlighted in the PLINK-WGAS illustrations (Figures 5 to 9); and their supportive biological importance was explained in the above sections. The other single SNPs with genomic control P values<0.01 which were not part of the associated genomic regions in our Bayesian analyses were not emphasized in a biological context in the above sections. Due to high computational requirements of haplotype construction, the haplotypes were only derived for the genomic regions found to be significant using the Bayesian approach. In addition to the consideration of single SNPs using the criteria of genomic control P values, the importance of haplotypes with genomic control P values<0.05 were explained in the above sections. Overall, the single SNP and haplotype analyses were supportive for the Bayesian approach results.

## Conclusions

The proportion of the phenotypic variance captured by markers across the genome was relatively high for RFI (0.52) compared to its related traits ADFI (0.47), ADG (0.34), and BF (0.49). Allele frequency differences at generation 8 identified that RFI selection lines likely differ in genes related to insulin and leptin regulation and in genes involved in metabolism in the liver and gastrointestinal tract. Although the posterior probabilities of association were modest, genomic selection based Bayesian methods were more powerful to detect associations by WGAS than frequentist in this data set. The WGAS revealed that genes involved in insulin release (e.g., *GLP1R, CDKAL, SGMS1*) partly explained variation in RFI and ADFI. Other energy homeostasis genes (e.g., *MC4R*, *PGM1* and *GPR81*) and muscle growth genes (e.g., *TGFB1*), were found to be associated with ADG. Genomic regions containing fat metabolism genes (e.g., *ACOXL*, *AEBP1*) and a gene for skeletal myogenesis (*KLHL31*) were associated with BF and LMA, respectively, in this population. Overall, the current study provided a list of genomic regions and candidate genes associated with RFI and its related traits for future validation studies in other populations prior to incorporation in marker assisted selection programs. Specifically, the study provided a very highly significantly associated QTL for LMA for fine mapping.

## Supporting Information

Figure S1 **Q-Q plots based on unadjusted P-values and corrected P-values using genomic control for the whole genome single SNP association analyses performed by the PLINK software.** The X and Yaxes represent expected and observed P-values, respectively. Population stratification represented by deviations of most of the unadjusted empirical P-values from expection was corrected by genomic control for all traits. The deviation of the two SNPs associated with LMA from the expectations after genomic control indicates that they are not likely to be false positives. RFI: Residual feed intake; ADFI: Average daily feed intake; ADG: Average daily gain; BF: Back fat; LMA: Loin muscle area.
(TIFF)

Figure S2 **Q-Q plots based on unadjusted P-values and corrected P-values using genomic control for the haplotype association analyses performed by the PLINK software.** The X and Yaxes represent expected and observed P-values, respectively. Population stratification represented by deviations of most of the unadjusted empirical P-values from expection was corrected by genomic control for all traits. The deviation of the two SNPs associated with LMA from the expectations after genomic control indicates that they are not likely to be false positives. RFI: Residual feed intake; ADFI: Average daily feed intake; ADG: Average daily gain; BF: Back fat; LMA: Loin muscle area.
(TIFF)

Table S1 **Posterior means of variance components explained by genome wide markers for RFI and its related traits using RFI selection lines by a Bayesian approach.**
(DOCX)

Table S2 **Detailed information about candidate QTL regions associated with the residual feed intake (RFI) by 1 Mb SNP window, single SNP and haplotype analyses.**
(DOCX)

Table S3 **Detailed information about candidate QTL regions associated with the average daily feed intake (ADFI) by 1 Mb SNP window, single SNP and haplotype analyses.**
(DOCX)

Table S4 **Detailed information about candidate QTL regions associated with the average daily gain (ADG) by 1 Mb SNP window, single SNP and haplotype analyses.**
(DOCX)

Table S5 **Detailed information about candidate QTL regions associated with the back fat (BF) by 1 Mb SNP window, single SNP and haplotype analyses.**
(DOCX)

Table S6 **Detailed information about candidate QTL regions associated with the loin muscle area (LMA) by 1 Mb SNP window, single SNP and haplotype analyses.**
(DOCX)

## References

[1] Boggess M (2009) A pork industry perspective. Pig Genome III Conference, Hinxton, UK, Nov. 2–4.

[2] Cai W, Casey DS, Dekkers JCM (2008) Selection response and genetic parameters for residual feed intake in Yorkshire swine. J Anim Sci 86: 287–298. 17998435 10.2527/jas.2007-0396

[3] Gilbert H, Bidanel JP, Gruand J, Caritez JC, Billon Y, et al (2007) Genetic parameters for residual feed intake in growing pigs, with emphasis on genetic relationships with carcass and meat quality traits. J Anim Sci 85: 3182–3188. 17785600 10.2527/jas.2006-590

[4] Hoque MA, Suzuki K (2009) Genetics of residual feed intake in cattle and pigs: A Review. Asian-Aust. J Anim Sci 22: 747–755.

[5] Hoque MA, Kadowaki H, Shibata T, Oikawa T, Suzuki K (2007) Genetic parameters for measures of the efficiency of gain of boars and the genetic relationships with its component traits in Duroc pigs. J Anim Sci 85: 1873–1879. 17431052 10.2527/jas.2006-730

[6] Johnson ZB, Chewning JJ, Nugent RA (1999) Genetic parameter for production traits and measures of residual feed intake in Large White swine. J Anim Sci 77: 1679–1685. 10438012 10.2527/1999.7771679x

[7] Mrode RA, Kennedy BW (1993) Genetic variation in measures of food efficiency in pigs and their genetic relationships with growth rate and backfat. Anim Prod 56: 225–232.

[8] Nguyen NH, McPhee CP, Wade CM (2005) Responses in residual feed intake in lines of Large White pigs selected for growth rate on restricted feeding (measured on *ad libitum* individual feeding). J Anim Breed Genet 122: 264–270. 16060494 10.1111/j.1439-0388.2005.00531.x

[9] Von Flede A, Roehe R, Looft H, Kalm E (1996) Genetic association between feed intake and feed intake behaviour at different stages of growth of group-housed boars. Livest Prod Sci 47: 11–22.

[10] Young JM, Cai W, Dekkers JCM (2011) Effect of selection for residual feed intake on feeding behavior and daily feed intake patterns in Yorkshire swine. J Anim Sci 89: 639–647. 21036935 10.2527/jas.2010-2892

[11] Onteru SK, Fan B, Nikkilä MT, Garrick DJ, Stalder KJ, et al (2011) Whole-genome association analyses for lifetime reproductive traits in the pig. J Anim Sci 89: 988–995. 21183715 10.2527/jas.2010-3236

[12] Onteru SK, Fan B, Du ZQ, Garrick DJ, Stalder KJ, et al (2012) A whole-genome association study for pig reproductive traits. Anim Genet 43: 18–26. 22221021 10.1111/j.1365-2052.2011.02213.x

[13] Fan B, Onteru SK, Du ZQ, Garrick DJ, Stalder KJ, et al (2011) Genome-wide association study identifies loci for body composition and structural soundness traits in pigs. PLoS ONE 6: e14726. 21383979 10.1371/journal.pone.0014726 PMC3044704

[14] Sherman EL, Nkrumah JD, Moore SS (2010) Whole genome single nucleotide polymorphism associations with feed intake and feed efficiency in beef cattle. J Anim Sci 88: 16–22. 19749024 10.2527/jas.2008-1759

[15] Barendse W, Reverter A, Bunch RJ, Harrison BE, Barris W, et al (2007) A validated whole-genome association study of efficient food conversion in cattle. Genetics 176: 1893–1905. 17507676 10.1534/genetics.107.072637 PMC1931545

[16] Rolf MM, Taylor JF, Schnabel RD, McKay SD, McClure MC, et al (2011) Genome-wide association analysis for feed efficiency in Angus cattle. Anim Genet 43: 367–374. 22497295 10.1111/j.1365-2052.2011.02273.x PMC3437496

[17] Nkrumah JD, Sherman EL, Li C, Marques E, Crews DH, et al (2007) Primary genome scan to identify putative quantitative trait loci for feedlot growth rate, feed intake, and feed efficiency of beef cattle. J Anim Sci 85: 3170–3181. 17709790 10.2527/jas.2007-0234

[18] Bolormaa S, Hayes BJ, Savin K, Hawken R, Barendse W, et al (2011) Genome-wide association studies for feedlot and growth traits in cattle. J Anim Sci 89: 1684–1697. 21239664 10.2527/jas.2010-3079

[19] Mujibi FDN, Nkrumah JD, Durunna ON, Stothard P, Mah J, et al (2011) Accuracy of genomic breeding values for residual feed intake in crossbred beef cattle. J Anim Sci 89: 3353–3361. 21642493 10.2527/jas.2010-3361

[20] Casey DS, Stern HS, Dekkers JCM (2005) Identifying errors and factors associated with errors in data from electronic swine feeders. J Anim Sci 83: 969–982. 15827241 10.2527/2005.835969x

[21] R Development Core Team (2011) R: A language and environment for statistical computing. R Foundation for Statistical Computing, Vienna, Austria. ISBN 3-900051-07-0. Available: http://www.R-project.org/. Accessed 2012 Nov 5.

[22] Bejamini Y, Hochberg Y (1995) Controlling the false discovery rate: a practical and powerful approach for multiple testing. J R Statist Soc B 57: 287–300.

[23] Meuwissen THE, Hayes BJ, Goddard ME (2001) Prediction of total genetic value using genome-wide dense marker maps. Genetics 157: 1819–1829. 11290733 10.1093/genetics/157.4.1819 PMC1461589

[24] Fernando RL, Garrick DJ (2008) GenSel—User manual for a portfolio of genomic selection related analyses. Animal Breeding and Genetics, Iowa State University, Ames. Available: http://bigs.ansci.iastate.edu/bigsgui/login.html. Accessed 2012 Sept 15.

[25] Habier D, Fernando RL, Kizilkaya K, Garrick DJ (2011) Extension of the Bayesian alphabet for genomic selection. BMC Bioinformatics 23: 186. 10.1186/1471-2105-12-186 PMC3144464 21605355

[26] Saatchi M, Garrick DJ, Fernando RL, Boddicker N (2012) Comparison of different Bayesian methods for QTL mapping in Hereford beef cattle using 1 Mb windows. Plant and Animal Genome Conference XX, Jan 14–18, San Diego, CA. P0552.

[27] Purcell S, Neale B, Todd-Brown K, Thomas L, Ferreira MAR, et al (2007) PLINK: a toolset for whole-genome association and population-based linkage analysis. Am J Hum Genet 81: 559–575 http://pngu.mgh.harvard.edu/purcell/plink/ (Accessed 2012 November 5^th^).. 17701901 10.1086/519795 PMC1950838

[28] Lee KT, Byun MJ, Kang KS, Park EW, Lee SH, et al (2011) Neuronal genes for subcutaneous fat thickness in human and pig are identified by local genomic sequencing and combined SNP association study. PLoS One 6: e16356. 21311593 10.1371/journal.pone.0016356 PMC3032728

[29] Barret JC, Fry B, Maller J, Daly MJ (2005) Haploview: analysis and visualization of LD and haplotype maps. Bioinformatics 21: 263–265 http://www.broad.mit.edu/mpg/haploview/ (Accessed 2012 October 15^th^ 15297300 10.1093/bioinformatics/bth457

[30] Stephens M, Scheet P (2005) Accounting for decay of linkage disequilibrium in haplotype inference and missing data imputation. Am J Hum Genet 76: 449–462. 15700229 10.1086/428594 PMC1196397

[31] Stephens M, Smith NJ, Donnelly P (2001) A new statistical method for haplotype reconstruction from population data. Am J Hum Genet 68: 978–989. 11254454 10.1086/319501 PMC1275651

[32] Zeng J (2011) Genomic selection of purebred animals for crossbred performance under dominance. A master's thesis submitted to Iowa State University.

[33] Okamoto K, Iwasaki N, Doi K, Noiri E, Iwamoto Y, et al (2012) Inhibition of glucose-stimulated insulin secretion by KCNJ15, a newly identified susceptibility gene for type 2 diabetes. Diabetes 61: 1734–1741. 22566534 10.2337/db11-1201 PMC3379671

[34] Kobayashi T, Zadravec D, Jacobsson A (2007) *ELOVL2* over expression enhances triacylglycerol synthesis in 3T3-L1 and F442A cells. FEBS Letters 581: 3157–3163. 17583696 10.1016/j.febslet.2007.05.081

[35] Friedman JM, Halaas JL (1998) Leptin and the regulation of body weight in mammals. Nature 395: 763–770. 9796811 10.1038/27376

[36] Schwartz MW, Woods SC, Porte D Jr, Seeley RJ, Baskin DG (2000) Central nervous system control of food intake. Nature 404: 661–671. 10766253 10.1038/35007534

[37] Woods SC, Lutz TA, Geary N, Langhans W (2006) Pancreatic signals controlling food intake; insulin, glucagon and amylin. Phil Trans R Soc B 361: 1219–1235. 16815800 10.1098/rstb.2006.1858 PMC1642707

[38] Li Q, Luo C, Löhr CV, Dashwood RH (2011) Activator protein-2a functions as a master regulator of multiple transcription factors in the mouse liver. Hepatol Res 41: 776–783. 21682828 10.1111/j.1872-034X.2011.00827.x PMC4139281

[39] Girard C, Duprat F, Terrenoire C, Tinel N, Fosset M, et al (2001) Genomic and functional characteristics of novel pancreatic 2P domain K(+) channels. Biochem Biophys Res Commun 282: 249–256. 11263999 10.1006/bbrc.2001.4562

[40] Drucker DJ, Philippe J, Mojsov S, Chicko WL, Habener JF (1987) Glucagon-like peptide I stimulates insulin gene expression and increases cyclic AMP levels in a rat islet cell line. Proc Nati Acad Sci USA 84: 3434–3438. 10.1073/pnas.84.10.3434 PMC304885 3033647

[41] Ohara-Imaizumi M, Yoshida M, Aoyagi K, Saito T, Okamura T, et al (2010) Deletion of *CDKAL1* affects mitochondrial ATP generation and first-phase insulin exocytosis. PLoS One 5: e15553. 21151568 10.1371/journal.pone.0015553 PMC3000340

[42] Le NT, Le FN, Louveau I, Gilbert H, Gondret F (2012) Metabolic changes and tissue responses to selection on residual feed intake in growing pigs. J Anim Sci [Epub ahead of print] 10.2527/jas.2012-5226 22871936

[43] Yuzaki M (2008) Cbln and C1q family proteins: new transneuronal cytokines. Cell Mol Life Sci 65: 1698–705. 18278437 10.1007/s00018-008-7550-3 PMC11131701

[44] Yano M, Watanabe K, Yamamoto T, Ikeda K, Senokuchi T, et al (2011) Mitochondrial dysfunction and increased reactive oxygen species impair insulin secretion in sphingomyelin synthase 1-null mice. J Biol Chem 286: 3992–4002. 21115496 10.1074/jbc.M110.179176 PMC3030399

[45] Strowski MZ, Kaczmarek P, Mergler S, Wiedenmann B, Domin D, et al (2009) Insulinostatic activity of cerebellin—Evidence from in vivo and in vitro studies in rats. Regul Pept 157 2009: 19–24. 19481574 10.1016/j.regpep.2009.05.010

[46] Sherman EL, Nkrumah JD, Moore SS (2010) Whole genome single nucleotide polymorphism associations with feed intake and feed efficiency in beef cattle. J Anim Sci 88: 16–22. 19749024 10.2527/jas.2008-1759

[47] Pal A, Barber TM, Van de Bunt M, Rudge SA, Zhang Q, et al (2012) PTEN mutations as a cause of constitutive insulin sensitivity and obesity. N Engl J Med 367: 1002–1011. 22970944 10.1056/NEJMoa1113966 PMC4072504

[48] Townsend KL, Suzuki R, Huang TL, Jing E, Schulz TJ, et al (2012) Bone morphogenic protein 7 (BMP7) reverses obesity and regulates appetite through a central mTOR pathway. FASEB J 26: 2187–2196. 22331196 10.1096/fj.11-199067 PMC3336788

[49] Begriche K, Marston OJ, Rossi J, Burke LK, McDonald P, et al (2012) Melanocortin-3 receptors are involved in adaptation to restricted feeding. Genes Brain Behav 11: 291–302.50. 22353545 10.1111/j.1601-183X.2012.00766.x PMC3319531

[50] Kim KS, Larsen N, Short T, Plastow G, Rothschild MF (2000) A missense variant of the porcine melanocortin-4 receptor *(MC4R)* gene is associated with fatness, growth, and feed intake traits. Mamm Genome 11: 131–135. 10656927 10.1007/s003350010025

[51] Piorkowska K, Tyra M, Rogoz M, Ropka-Molik K, Oczkowicz M (2010) Association of the melanocortin-4 receptor (MC4R) with feed intake, growth, fatness and carcass composition in pigs raised in Poland. Meat Sci 85: 297–301. 20374902 10.1016/j.meatsci.2010.01.017

[52] Seeley RJ, Yagaloff KA, Fisher SL, Burn P, Thiele TE, et al (1997) Melanocortin receptors in leptin effects. Nature 390: 349. 10.1038/37016 9389472

[53] Obici S, Feng Z, Tan J, Liu L, Karkanias G, et al (2001) Central melanocortin receptors regulate insulin action. J Clin. Invest 108: 1079–1085. 11581309 10.1172/JCI12954 PMC200952

[54] Flock GB, Cao X, Maziarz M, Drucker DJ (2012) Activation of enteroendocrine membrane progesterone receptors promotes incretin secretion and improves glucose tolerance in mice. Diabetes [Epub ahead of print] 10.2337/db12-0601 PMC3526055 22933106

[55] Van den Maagdenberg K, Stinckens A, Claeys E, Seynaeve M, Clinquart A, et al (2007) The Asp298Asn missense mutation in the porcine melanocortin-4 receptor (MC4R) gene can be used to affect growth and carcass traits without an effect on meat quality. Animal 1: 1089–1098. 22444853 10.1017/S1751731107000456

[56] Kim KS, Lee JJ, Shin HY, Choi BH, Lee CK, et al (2006) Association of melanocortin 4 receptor (MC4R) and high mobility group AT-hook 1 (HMGA1) polymorphisms with pig growth and fat deposition traits. Anim Genet 37: 419–421. 16879362 10.1111/j.1365-2052.2006.01482.x

[57] Davoli R, Braglia S, Valastro V, Annarratone C, Comella M, et al (2012) Analysis of MC4R polymorphism in Italian Large White and Italian Duroc pigs: Association with carcass traits. Meat Sci 90: 887–892. 22197097 10.1016/j.meatsci.2011.11.025

[58] Liu W, Yan M, Liu Y, Wang R, Li C, et al (2010) Olfactomedin 4 down-regulates innate immunity against *Helicobactor pylori* infection. Proc Natl Acad Sci USA 107: 11056–11061. 20534456 10.1073/pnas.1001269107 PMC2890768

[59] Bradfield JP, Taal HR, Timpson NJ, Scherag A, Lecoeur C, et al (2012) A genome-wide association meta-analysis identified new childhood obesity loci. Nature Genet 44: 526–531. 22484627 10.1038/ng.2247 PMC3370100

[60] Kim HR, Ingham PW (2009) The extracellular matrix protein TGFBI promotes myofibril bundling and muscle fibre growth in the Zebrafish embryo. Dev Dyn 238: 56–65. 19097068 10.1002/dvdy.21812

[61] Lin CS, Hsu CW (2005) Differentially transcribed genes in skeletal muscle of Duroc and Taoyuan pigs. J Anim Sci 83: 2075–2086. 16100062 10.2527/2005.8392075x

[62] Cai TQ, Ren N, Jin L, Cheng K, Kash S, et al (2008) Role of GPR81 in lactate-mediated reduction of adipose lipolysis. Biochem and Biophys Res Commun 377: 987–991. 18952058 10.1016/j.bbrc.2008.10.088

[63] Takeuchi K, Reue K (2009) Biochemistry, physiology, and genetics of GPAT, AGPAT, and lipin enzymes in triglyceride synthesis. Am J Physiol Endocrinol Metab 296: E1195–E1209. 19336658 10.1152/ajpendo.90958.2008 PMC2692402

[64] Clemente-Postigo M, Queipo-Ortun MI, Fernandez-Garcia D, Gomez-Huelgas R, Tinahones FJ, et al (2011) Adipose tissue gene expression of factors related to lipid processing in obesity. PLoS One 6: e24783. 21966368 10.1371/journal.pone.0024783 PMC3178563

[65] Damon M, Louveau I, Lefaucheur L, Lebret B, Vincent A, et al (2006) Number of intramuscular adipocytes and fatty acid binding protein-4 content are significant indicators of intramuscular fat level in crossbred Large White×Duroc pigs. J Anim Sci 84: 1083–1092. 16612010 10.2527/2006.8451083x

[66] Abou-Elhamd A, Cooper O, Münsterberg A (2009) Klhl31 is associated with skeletal myogenesis and its expression is regulated by myogenic signals and Myf-5. Mech Dev 126: 852–862. 19643178 10.1016/j.mod.2009.07.006

[67] Jung YC, Rothschild MF, Flanagan MP, Warner CM, Christian LL (1989) Association of restriction fragment length polymorphisms of swine leucocyte antigen class I genes and production traits in Duroc and Hampshire boars. Anim Genet 20: 79–91. 2567137 10.1111/j.1365-2052.1989.tb00845.x

[68] Hoff PD, (2009) A First Course in Bayesian Statistical Methods. New York, Springer Press. 5p.

[69] Meuwissen TH, Hayes BJ, Goddard ME (2001) Prediction of total genetic value using genome-wide dense marker maps. Genetics 157: 1819–1829. 11290733 10.1093/genetics/157.4.1819 PMC1461589

