# Supplementary figures and images for: Whole Genome Association Studies of Residual Feed Intake and Related Traits in the Pig
Source: PLoS One. 2013 Jun 26;8(6):e61756. doi: 10.1371/journal.pone.0061756 (PMC3694077; doi:10.1371/journal.pone.0061756)

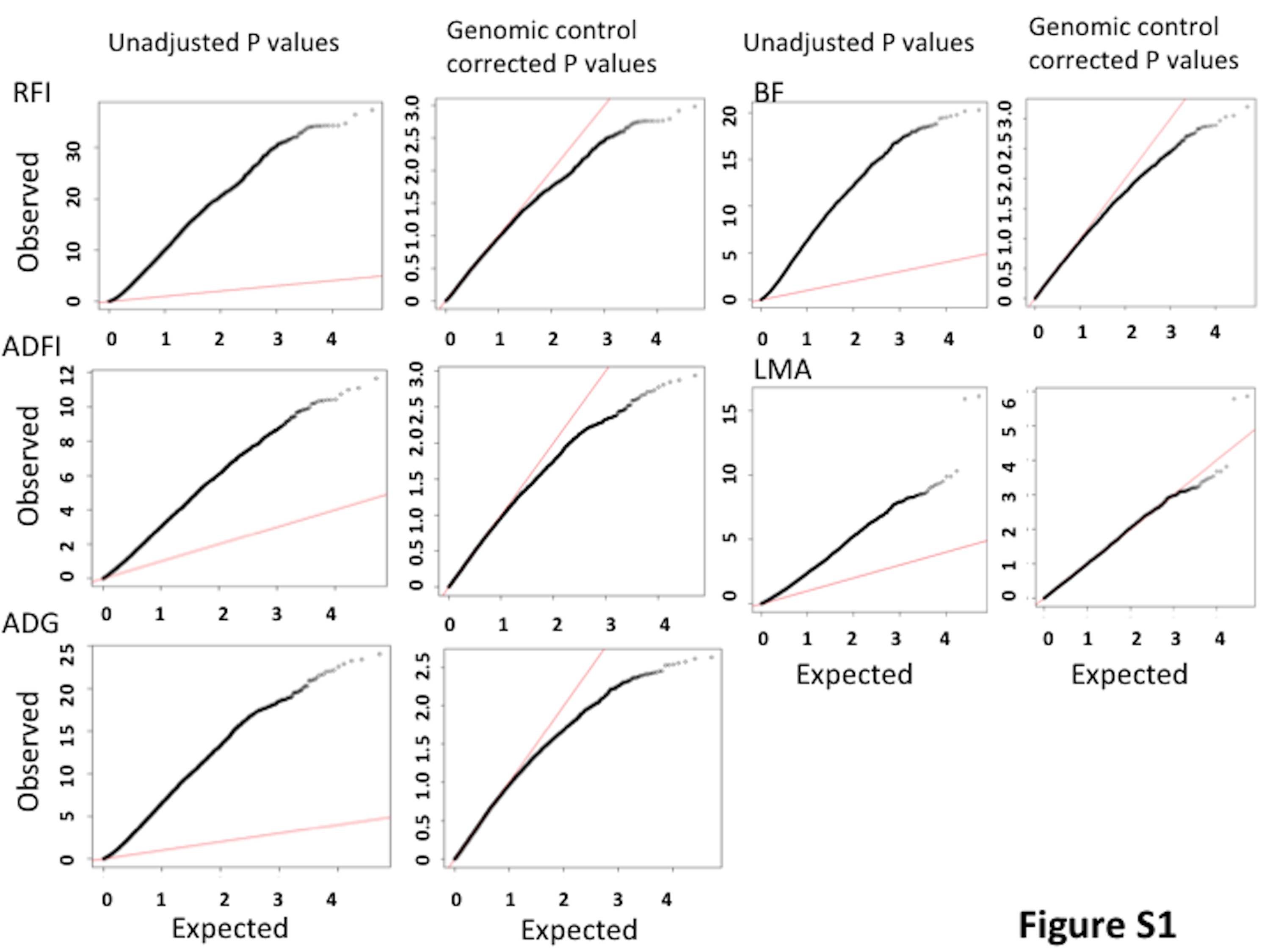

Supplement: Figure S1 — Q-Q plots based on unadjusted P-values and corrected P-values using genomic control for the whole genome single SNP association analyses performed by the PLINK software. The X and Yaxes represent expected and observed P-values, respectively. Population stratification represented by deviations of most of the unadjusted empirical P-values from expection was corrected by genomic control for all traits. The deviation of the two SNPs associated with LMA from the expectations after genomic control indicates that they are not likely to be false positives. RFI: Residual feed intake; ADFI: Average daily feed intake; ADG: Average daily gain; BF: Back fat; LMA: Loin muscle area. (TIFF) [file pone.0061756.s001.tiff]

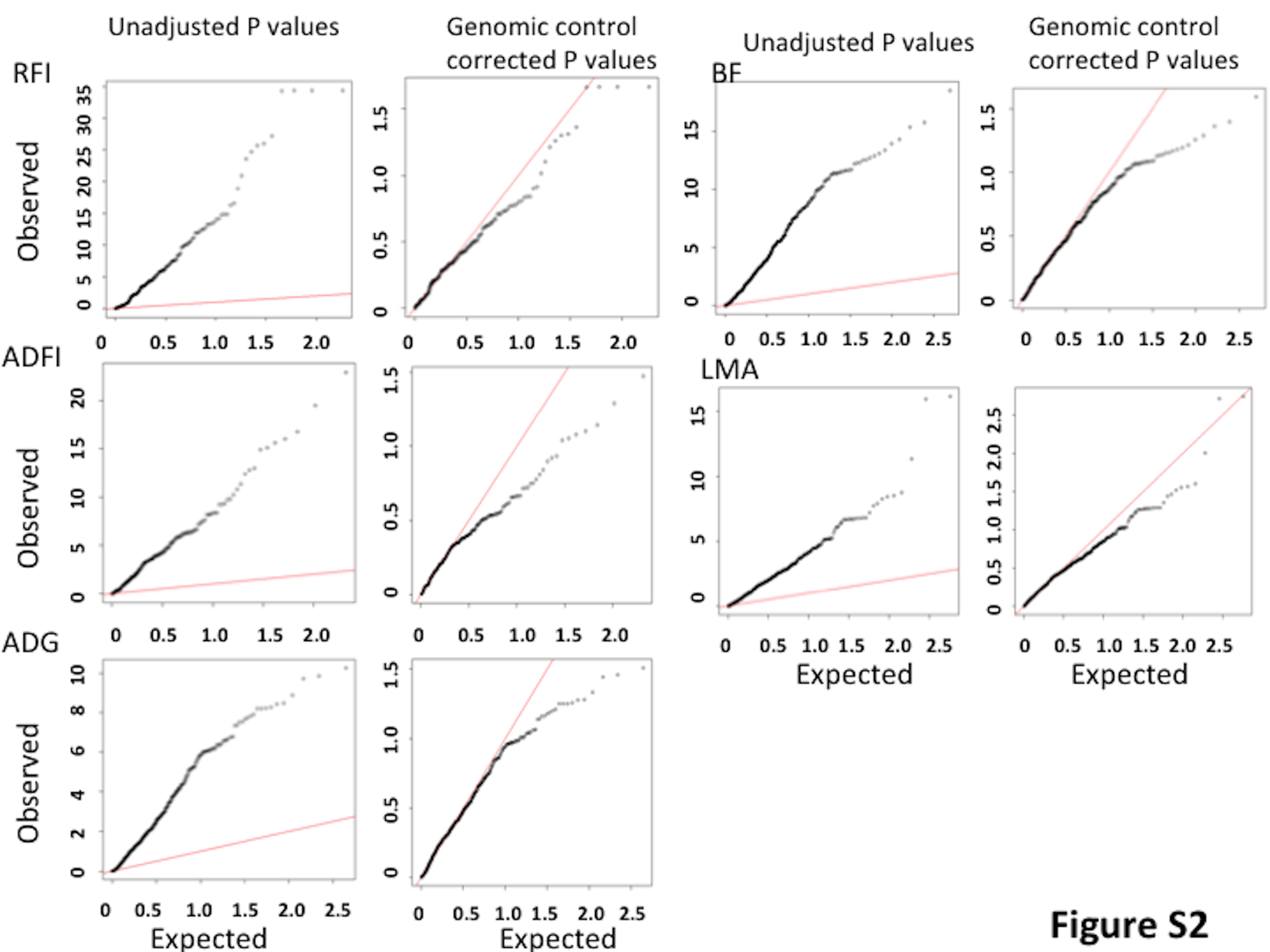

Supplement: Figure S2 — Q-Q plots based on unadjusted P-values and corrected P-values using genomic control for the haplotype association analyses performed by the PLINK software. The X and Yaxes represent expected and observed P-values, respectively. Population stratification represented by deviations of most of the unadjusted empirical P-values from expection was corrected by genomic control for all traits. The deviation of the two SNPs associated with LMA from the expectations after genomic control indicates that they are not likely to be false positives. RFI: Residual feed intake; ADFI: Average daily feed intake; ADG: Average daily gain; BF: Back fat; LMA: Loin muscle area. (TIFF) [file pone.0061756.s002.tiff]
