# Supplementary material for: Whole Genome Association Studies of Residual Feed Intake and Related Traits in the Pig
Source: PLoS One. 2013 Jun 26;8(6):e61756. doi: 10.1371/journal.pone.0061756 (PMC3694077; doi:10.1371/journal.pone.0061756)
Supplement: Table S1 — Posterior means of variance components explained by genome wide markers for RFI and its related traits using RFI selection lines by a Bayesian approach. (DOCX) [file pone.0061756.s003.docx]

**Table S1. Posterior means of variance components explained by genome wide markers for RFI and its related traits using RFI selection lines by a Bayesian approach.**

| **Trait** | **No. of animals** | **Genetic variance** | **Residual variance** | **Estimated total variance** | **Proportion of phenotypic variance explained by markers** |
| --- | --- | --- | --- | --- | --- |
| RFI | 1410 | 0.014 | 0.013 | 0.027 | 0.523 |
| ADFI | 1417 | 0.021 | 0.023 | 0.044 | 0.475 |
| ADG | 1418 | 0.002 | 0.004 | 0.006 | 0.341 |
| BF | 1412 | 6.097 | 6.322 | 12.419 | 0.490 |
| LMA | 1410 | 7.733 | 12.345 | 20.078 | 0.385 |
