# Supplementary material for: Whole Genome Association Studies of Residual Feed Intake and Related Traits in the Pig
Source: PLoS One. 2013 Jun 26;8(6):e61756. doi: 10.1371/journal.pone.0061756 (PMC3694077; doi:10.1371/journal.pone.0061756)
Supplement: Table S2 — Detailed information about candidate QTL regions associated with the residual feed intake (RFI) by 1 Mb SNP window, single SNP and haplotype analyses. (DOCX) [file pone.0061756.s004.docx]

**Table S2. Detailed information about candidate QTL regions associated with the residual feed intake (RFI) by 1Mb SNP window, single SNP and haplotype analyses.**

| SSC | Location (start-end) in Mb^@^ | 1Mb SNP window | Percent genetic variance explained | PPA* (P > 0) | Genes within the SNP window^$^ | Genes within 1Mb upstream the SNP window^$^ | Genes within 1Mb downstream the SNP window^$^ | Previously reported important QTL at the SNP window | Associated single SNPs (position in Mb) within the 1Mb window** | Associated haplotypes within the 1Mb window*** |
| --- | --- | --- | --- | --- | --- | --- | --- | --- | --- | --- |
| 14 | 59.00 - 59.98 | H3GA0040291-MARC0009335 | 1.16 | 0.682 | *GPR137, LYST, GNG4, B3GALN, TBCE* | *MTR, ACTN2, HEATR1, LGALS8, EDARAD, Y-RNA* | *SNORA18, SNORA14, GGPS1, ARID4B, RBM34, TOMM20, TARBP1, C1ORF31, SLC35F3* | Daily feed intake and body weight | - | - |
| 7 | 39.01 - 39.98 | ALGA0040519-ASGA0032851 | 0.59 | 0.480 | *BTBD9, GLO1, DNAH8, GLP1R, KCNK5, KCNK17* | *MDGA1, ZFAND3, BTBD9* | *U6, KIF6, DAAM2, MOCS1, LRFN2* | Average daily gain and body weight | H3GA0020982 (39.53), ALGA0040598 (39.90) | DIAS0000368 (G) - H3GA0020971 (T) - ALGA0040539 (A) - M1GA0010069 (T) - ASGA0032785 (A) - H3GA0020982 (T) - H3GA0020988 (T) - ALGA0040570 (T) |
| 7 | 16.06 - 16.97 | ALGA0038863-DRGA0007204 | 0.45 | 0.412 | *ID4, MBOAT1, E2F3, CDKAL1* | No annotated genes | *SOX4, CDKAL1* | Average daily gain and body weight | - | CASI0009829 (G) - ASGA0031485 (G) - ALGA0038950 (G) - ASGA0031489 (C) - ALGA0038953 (G) - DRGA0007199 (T) - DRGA0007201 (A) - MARC0037047 (A) |
| 14 | 90.03 - 90.96 | ASGA0064826-ALGA0079379 | 0.40 | 0.336 | *5S - rRNA* | *SH2D4B* | *U6, SNORA31, NRG3, Novel miRNA* | Average daily gain and body weight | MARC0041088 (90.28), ASGA0064841 (90.33), ASGA0064844 (90.36), ALGA0079359 (90.45), ASGA0064847 (90.48), ASGA0064852 (90.58), MARC0088303 (90.79), DRGA0014176 (90.82), DRGA0014177 (90.86), ALGA0079375 (90.92) | Haplotype 1 MARC0038712 (C) - MARC0041088 (T) - DRGA0014176 (A) - DRGA0014177 (A) - ALGA0079375 (G); Haplotype 2 ASGA0064824 (C) - ALGA0079331 (G) - ASGA0064826 (T) - MARC0073576 (C); Haplotype 3 H3GA0041215 (A) - ALGA0079341 (A) - ALGA0079343 (A) - INRA0045306 (G) - ALGA0079346 (T) - ALGA0079348 (C) - MARC0041088 (T) - ALGA0079351 (C) - ASGA0064841 (G) - ASGA0064844 (T). |
| 2 | 111.01 - 111.94 | MARC0103716-DRGA0003323 | 0.33 | 0.326 | *SLCO4C1* | *FAM174A, ST8SIA4* | *SNORD112, 5S_rRNA, C5orf30, SLCO4C1, SLCO6A1, PAM, GIN1* | Average daily gain and body weight | DRGA0003319 (111.51) | - |
| 14 | 4.11 – 4.99 | ALGA0074381-H3GA0038441 | 0.32 | 0.340 | *SLC18A1, ATP6V1B2, LZTS1* | *AUH, NFIL3, ROR2, SPTLC1* | *SNORA25* | No reported QTL | - | - |
| 9 | 13.00 - 13.97 | H3GA0026577-ALGA0051489 | 0.31 | 0.340 | *PAK1, AQP11, CLNS1A, RSF-1, KCTD14, THRSP, NDUFC2, ALG8, C11ORF67, INTS4* | *TSKU, ACER3, FTH1, ACER3, B3GNT6, CAPN5, MYO7A, LOC100524885, GDPD4* | *GAB2, NARS2* | Average daily feed intake and body weight | - | - |
| 5 | 20.01 - 20.95 | ALGA0031069-ALGA0031122 | 0.30 | 0.411 | *HOXC5,MIR615, HOXC4, Novel protein coding, HNRNPA1L2, NFE2, COPZ1, MIR148B, pseudogene, ZNF385A, ITGA5, GTSF1, PDE1B, NCKAP1L, KIAA0748, U6, NEUROD4* | *MIR615, U6, ATP5G2, CALCOCO1, ATP5G2, HOXC12, HOXC13, HOXC10, HOXC8, HOXD11, HOXC5, HOXC6, SP1, AMHR2, PRR13, PCBP2, MAP3K12, TARBP2, NPFF, ATF7* | *OR10C1, OR6C3, OR6C4, OR6C65, OR6C76, OR6C6* | Average daily gain and body weight | - | - |
| 14 | 2.03 - 2.99 | ASGA0060441-ALGA0074320 | 0.30 | 0.299 | *SH2D4B* | *SECISBP2, SEMA4D, GADD45G* | *AUH, NFIL3, ROR2, SPTLC1* | No reported QTL | - |  |
| 14 | 89.01 - 89.67 | ALGA0079296-ALGA0079331 | 0.28 | 0.262 | *SCD5, snRNA, Novel protein - coding, ENOPH1, HNRPDL, HNRNPD, RASGEF1B* | *PPIF, ANXA11, PLAC9, C10orf57, SFTPD, MBL1, SFTPA1, MAT1A, DYDC1, DYDC2, C10ORF58, TSPAN14, SH2D4B, ZMIZ1* | *5S_rRNA* | Average daily feed intake | MARC0057510 (89.05), MARC0029597 (89.07), ASGA0064809 (89.17), MARC0075340 (89.43), DRGA0014154 (89.49), ALGA0079330 (89.52) | MARC0057510 (A) - MARC0029597 (T) - ALGA0079307 (G) - ALGA0079308 (T) - ASGA0064809 (T) - ASGA0064811 (G) - H3GA0041207 (G) - ALGA0079318 (A) - MARC0027659 (G) - H3GA0041209 (A) - ASGA0064820 (C) - MARC0075340 (G) - ASGA0064823 (C) - DRGA0014154 (A) - ALGA0079330 (C) |
| 8 | 145.00 - 145.97 | ALGA0103902-MARC0034664 | 0.27 | 0.396 | *SCD5, snRNA, Novel protein - coding, ENOPH1, HNRPDL, HNRNPD, RASGEF1B* | *AGPAT9, FAM175A, HELQ, HPSE, COQ2, PLAC8, COPS4, LIN54, THAP9, SEC31A, SCD5* | *PRKG2, BMP3, C4orf22, FGF5, PRDM8, ANTXR2, HMGB3, IFI44* | Weight of ovary | - | - |
| 17 | 62.01 - 62.97 | M1GA0022361-DRGA0016829 | 0.27 | 0.319 | *DOK5* | *U6atac, 7SK, TSHZ2, PFDN4, ZNF217, BCAS1, CYP24A1* | *U6, CBLN4* | Body weight | M1GA0022361 (62.01) | - |
| 3 | 19.01 - 19.95 | ASGA0099267-ALGA0017911 | 0.25 | 0.353 | *XPO6, GSG1L, pseudogene, KIAA0556, GTF3C1, IL21R, IL4R, JMJD5* | *SNORA30, SNORD112, C16orf54, ZNF629, RNF40, C16orf93,PHKG2, SRCAP, FBRS, PRR14, ZNF689, ZNF764, ITGAL, ZNF768, DCTPP1, SEPHS2, ZNF48, SEPT1, HUMMLC2B, TBC1D10B, CD2BP2, SEZ6L2, CDIPT, MVP, C16orf53, PRRT2, MAZ, KIF22, CD43, KCTD13, ASPHD1, ATXN2L, TUFM, SH2B1, ATP2A1, RABEP2, CD19, NFATC2IP, SPNS1, LAT, SBK1, XPO6* | *U6* | Average daily gain and body weight | - | ASGA0095600 (A) - ASGA0013765 (T) |
| 8 | 16.01 - 16.90 | DRGA0008347-MARC0043725 | 0.25 | 0.307 | *GPR125* | *SLIT2, PACRGL, KCNIP4, PERV* | *GPR125, GBA3* | Average daily gain and body weight | - | - |
| 9 | 135.02 - 135.89 | MARC0005452-ALGA0055107 | 0.25 | 0.403 | *CACNA1E, miRNA, ZNF648* | *XPR1, KIAA1614, STX6, MR1* | *U6, TEDDM1, GLUL, RGSL1, RGS16, RNASEL, RGS8, DHX9, LAMC1, LAMC2, NMNAT2* | Average daily gain and body weight | - | - |
| 15 | 52.00 - 52.92 | ALGA0085205-ALGA0085223 | 0.23 | 0.309 | *ENPP6, IRF2, CASP3, MLF1IP, protein coding, FACL2* | *CLDN22, STOX2, DCTD, WWC2, CDKN2AIP, ING2, RWDD4, C4ORF41* | *SNORA31, SLC25A4, KIAA1430, SNX25, LRP2BP, UFSP2, C4ORF47, CCDC110, PDLIM3, ANKRD37, SORBS2, CYP4V2, FAM149A, TLR3, KLK3* | body weight | - | - |
| 8 | 17.00 - 17.88 | ALGA0119511-ALGA0046694 | 0.20 | 0.263 | *Protein - coding* | *GPR125* | *DHX15* | Average daily gain and body weight | - | - |

^@^ The 1Mb windows are presented in descending order based on the percent genetic variance explained greater than 0.2%.

*Posterior probability that the SNPs in 1Mb window could explain the genetic variance greater than zero (PPA: Posterior probability of association).

**Association of single SNPs was considered based on genomic control corrected P-values at a threshold of 0.01 by the PLINK software

***Association of haplotypes was considered based on genomic control corrected P-values at a threshold of 0.05 by the PLINK software

^$^ The genes and their abbreviations are based on *Sus scrofa* genome build 10.2 and the BLAST analyses by the NCBI software.

Note: The windows with unmapped SNPs are not real consecutive SNP windows and hence they are not presented
