# Supplementary material for: Whole Genome Association Studies of Residual Feed Intake and Related Traits in the Pig
Source: PLoS One. 2013 Jun 26;8(6):e61756. doi: 10.1371/journal.pone.0061756 (PMC3694077; doi:10.1371/journal.pone.0061756)
Supplement: Table S3 — Detailed information about candidate QTL regions associated with the average daily feed intake (ADFI) by 1 Mb SNP window, single SNP and haplotype analyses. (DOCX) [file pone.0061756.s005.docx]

**Table S3. Detailed information about candidate QTL regions associated with the average daily feed intake (ADFI) by 1Mb SNP window, single SNP and haplotype analyses.**

| SSC | Location (start-end) in Mb^@^ | 1Mb SNP window | Percent genetic variance explained | PPA *(P > 0) | Genes within the SNP window^$^ | Genes within 1Mb upstream the SNP window^$^ | Genes within 1Mb downstream the SNP window^$^ | Previously reported important QTL at the SNP window | Associated single SNPs (position in Mb) within the 1Mb window** | Associated haplotypes within the 1Mb window*** |
| --- | --- | --- | --- | --- | --- | --- | --- | --- | --- | --- |
| 14 | 107.05 - 107.99 | ASGA0065520 - ALGA0080315 | 0.89 | 0.574 | *snoRNA, protein - coding, A1CF, ASAH2, SGMS1* | *DKK1, PRKG1, CSTF2T* | *SGMS1, MINPP1,PAPSS2, ATAD1, PTEN, U6ATAC* | Average daily feed intake, body weight and average daily gain | - | - |
| 17 | 63.00 - 63.97 | ALGA0096110 -M1GA0022378 | 0.86 | 0.538 | *miRNA, CBLN4, U6* | *CR956648.2, DOK5, CR956648.3* | *CR956640.5, CH242-266P8.1, MC3R, FAM210B, STK6, CSTF1, CASS4, C20orf43, GCNT7, C17H20orf106, TFAP2C, BMP7, SPO11, RAE1, RNPC1, U6* | Body weight (weaning) | - | - |
| 14 | 61.11 - 61.93 | MARC0044077 - ALGA0077929 | 0.79 | 0.469 | *KCNK1, protein - coding, PCNXL2* | *ARID4B, RBM34, TOMM20, TARBP1, C1orf31, SLC35F3, SNORA18, SNORA14* | *PCNXL2, KIAA1383, SIPA1L2, U6, SNORA70* | Feed intake, average daily gain and body weight | - | - |
| 1 | 177.00 - 177.96 | ALGA0006599 - INRA0004954 | 0.77 | 0.299 | *RNF152, CDH20, 7SK, MC4R (<0.1Mb)* | *PHLPP1, ZCCHC2, TNFRSF11A, KIAA1468, PIGN* | *MC4R, PMAIP1, CCBE1* | Daily feed intake and body weight | - | - |
| 14 | 59.00 -59.98 | H3GA0040291 - MARC0009335 | 0.68 | 0.374 | *GPR137B, LYST, GNG4, B3GALNT2, TBCE* | *LGALS8, EDARADD* | *GGPS1, ARID4B, RBM34, TOMM20, TARBP1, C1orf31, SLC35F3, SNORA18, SNORA14* | Daily feed intake and body weight | - | - |
| 1 | 172.01 -172.96 | MARC0076018 - ALGA0116832 | 0.51 | 0.257 | *U6, CDH19* | *5S_rRNA* | *CDH19, CDH7* | Feed intake, body weight and average daily gain | - | - |
| 6 | 135.07 -135.95 | INRA0022506 - H3GA0056466 | 0.47 | 0.425 | *LEPROT, DNAJC6, AK3L1, 5S - rRNA, ssc-mir-101a-2, miRNA, JAK1* | *SERBP1, IL12RB2, IL23RA, C1orf141, PDE4B* | *RAVER2, CACHD1, UBE2U* | Feed intake, body weight and average daily gain | - | - |
| 14 | 126.01 -126.98 | ALGA0081288 - ALGA0081327 | 0.47 | 0.287 | *SORCS3, U8* | *ITPRIP, CCDC147, WDR96, GSTO1* | *SORCS1* | Feed intake, body weight and average daily gain | ALGA0081306 (126.38) | ALGA0081306 (C) - MARC0041971 (G) - ALGA0081309 (A) - ALGA0081313 (G) |
| 4 | 138.05 -138.98 | ALGA0029412 - ASGA0023307 | 0.45 | 0.483 | *ZNF326,7SK, protein - coding* | *HFM1, ZNF644, BARHL2* | *PKN2, LRRC8C, GBP4, GBP6, GBP5, GBP6, GBP1* | Meat Quality | ALGA0029412 (138.05) | - |
| 14 | 15.020 -15.99 | ASGA0061613 - MARC0114177 | 0.44 | 0.361 | *MSRA,protein - coding, PRSS55, RP1L1, SOX7, PINX1, XKR6, miRNA, U6, BLK* | *KIF13B, MSRA, HMBOX1, ssc-mir-124a-2* | *GATA4, NEIL2, FDFT1, CTSB, DEFB134, ADAM20, ADAM29, GLRA3, HPGD, CEP44, FBXO8, RNaseP_nuc* | Average daily gain, feed intake and body weight | - | - |
| 11 | 28.01 - 28.99 | H3GA0031714 - ALGA0061627 | 0.43 | 0.354 | *U6 SnRNA* | *OLFM4* | *U6, MiRNA* | Meat quality | - | - |
| 1 | 176.01 -176.94 | INRA0004873 - ASGA0004980 | 0.42 | 0.215 | *PHLPP1, ZCCHC2, miRNA, TNFRSF11A, KIAA1468, PIGN* | *SERPINB8, SERPINB10, SERPINB11, SERPINB7, SERPINB2, VPS4B, SERPINB5, BCL2, KDSR, MiRNA, U6* | *RNF152, CDH20, 7SK* | Average daily gain, feed intake and body weight | - | - |
| 14 | 29.00 -29.98 | MARC0020707 - H3GA0039591 | 0.37 | 0.388 | *miRNA, TMEM132B, AACS, BRI3BP, DHX37, UBC* | *TMEM132C* | *SCARB1, FAM101A, ZNF664, DNAH10, ATP6V0A2, NCOR2, CCDC92* | Daily weight gain and body weight | INRA0043334 (29.32), ASGA0062403 (29.27), INRA0043333 (29.29) | - |
| 1 | 185.04 -185.89 | ALGA0006854 - H3GA0003303 | 0.35 | 0.265 | *GLCE, PAQR5, KIF23, RPLP1, protein - coding, TLE* | *ITGA11, CORO2B, AN32A, NOX5, ANP32A, GLCE, 7SK* | *UACA, LARP6, SNORA70, U6, TLE3, EGFL8, LRRC49* | Feed intake, Daily weight gain and body weight | MARC0088780 (185.75), ALGA0006895 (185.78), ALGA0006854 (185.04) | MARC0088780 (C) - ASGA0005234 (T) - ALGA0006895 (T) |
| 4 | 83.04 -83.84 | ASGA0020351 - ASGA0020384 | 0.34 | 0.294 | *protein - coding, RP1* | *IMPAD1, PENK, SDR16C5, CHCHD7, PLAG1, MOS, RPS20, LYN, TGS1, TMEM68, snoU54* | *MRPL15, LYPLA1, TCEA1, RGS20, ATP6V1H, OPRK1, NPBWR1, SOX17, 5S_rRNA* | Average daily gain, and body weight | - | - |
| 7 | 124.01 -124.91 | M1GA0010933 - ALGA0045373 | 0.33 | 0.356 | *TCL1B, TCL1A, BDKRB2, protein - coding, BDKRB1, ATG2B, C14ORF129, AK7, PAPOLA* | *SERPINA3-6, SERPINA3-2, Pseudogene, GSC, DICER1, CLMN, SYNE3, GLRX5, SCARNA13, SNORA31* | *AK7, PAPOLA, VRK1* | Body weight | ALGA0045338 (124.47)  ALGA0045326 (124.41) | - |
| 8 | 30.01 - 30.95 | ALGA0047182 - ALGA0047284 | 0.32 | 0.308 | *KIAA1239, C4ORF19, RELL1, PGM2, TBC1D1, protein - coding* | *ARAP2, DTHD1, CH242-305H5.1* | *KLF3, TLR10, TLR1, TLR6, FAM114A1, TBC1D1, TLR1* | Average daily gain and lipid accretion rate | - | - |
| 15 | 60.00 - 60.98 | H3GA0044381 - DRGA0015151 | 0.30 | 0.274 | *NRG1, protein - coding, U6* | *DUSP26, RNF122, TTI2, MAK16, HNRNPA2B1, NRG1, snoU13, U6* | *WRN, PURG, TEX15, PPP2CB, GSR, GTF2E2, RBPMS, U6, UBXN8* | Body weight | MARC0073466 (60.33), INRA0049417 (60.35) | - |
| 1 | 167.09 -167.88 | MARC0096493 - MARC0054709 | 0.27 | 0.141 | *miRNA, snRNA, CBLN2* | *NETO1* | No annotated genes | Average daily gain, feed intake and body weight | - | - |
| 14 | 56.08 - 56.74 | DRGA0013830 - ALGA0077635 | 0.27 | 0.179 | *No annotated genes* | *Psuedogene, RANBP1, ZDHHC8, RGD1563782, RTN4R, Ralgds, C22orf25, ssc-mir-1306, TRMT2A, MiRNA, ssc-mir-185, SNORA77* | *ZP4, RYR2* | Daily feed intake and body weight |  | - |

^@^ The 1Mb windows are presented in descending order based on the percent genetic variance explained greater than 0.2%.

*Posterior probability that the SNPs in 1Mb window could explain the genetic variance greater than zero (PPA: Posterior probability of association).

**Association of single SNPs was considered based on genomic control corrected P-values at a threshold of 0.01 by the PLINK software

***Association of haplotypes was considered based on genomic control corrected P-values at a threshold of 0.05 by the PLINK software

^$^ The genes and their abbreviations are based on *Sus scrofa* genome build 10.2

Note: The windows with unmapped SNPs are not real consecutive SNP windows and hence they are not presented
