# Supplementary material for: Whole Genome Association Studies of Residual Feed Intake and Related Traits in the Pig
Source: PLoS One. 2013 Jun 26;8(6):e61756. doi: 10.1371/journal.pone.0061756 (PMC3694077; doi:10.1371/journal.pone.0061756)
Supplement: Table S4 — Detailed information about candidate QTL regions associated with the average daily gain (ADG) by 1 Mb SNP window, single SNP and haplotype analyses. (DOCX) [file pone.0061756.s006.docx]

**Table S4. Detailed information about candidate QTL regions associated with the average daily gain (ADG) by 1Mb SNP window, single SNP and haplotype analyses.**

| SSC | Location (start-end) in Mb^@^ | 1Mb SNP window | Percent genetic variance explained | PPA* (P > 0) | Genes within the SNP window^$^ | Genes within 1Mb upstream the SNP window^$^ | Genes within 1Mb downstream the SNP window^$^ | Previously reported important QTL at the SNP window | Associated single SNPs (position in Mb) within the 1Mb window** | Associated haplotypes within the 1Mb window*** |
| --- | --- | --- | --- | --- | --- | --- | --- | --- | --- | --- |
| 1 | 177.00 – 177.96 | ALGA0006599 - INRA0004954 | 2.40 | 0.427 | *RNF152, CDH20, 7SK,* | *PHLPP1, ZCCHC2, TNFRSF11A, KIAA1468, PIGN* | *MC4R (<0.1Mb), PMAIP1, CCBE1* | Average daily gain, feed intake and body weight | ALGA0006599 (177.00), ALGA0006602 (177.12), DIAS0000206 (177.33), ASGA0004988 (177.53), ASGA0004990 (177.56), ASGA0004992 (177.74) | ALGA0006612 (C) - ASGA0004992 (A) - ASGA0004994 (G) - ALGA0006619 (G) - ALGA0006621 (G) - ASGA0004998 (G) - INRA0004954 (A) |
| 11 | 28.01 – 28.99 | H3GA0031714 - ALGA0061627 | 1.94 | 0.664 | U6 | *OLFM4* | *U6, miRNA* | Meat quality | - | - |
| 1 | 176.01 – 176.94 | INRA0004873 - ASGA0004980 | 1.56 | 0.304 | *PHLPP1, ZCCHC2, miRNA, TNFRSF11A, KIAA1468, PIGN* | *SERPINB8, SERPINB10, SERPINB11, SERPINB7, SERPINB2, VPS4B, SERPINB5, BCL2, KDSR, U6* | *RNF152, CDH20, 7SK* | Average daily gain, feed intake and body weight | ASGA0004970 (176.06), ALGA0006582 (176.12), MARC0034873 (176.15), INRA0004895 (176.23), MARC0075909 (176.70) | - |
| 2 | 143.05 – 143.91 | ASGA0098016 - ASGA0085390 | 1.02 | 0.532 | *H2AFY, NEUROG1, CXCL14, SLC25A48, IL9, protein - coding, LECT2, TGFBI, Vault* | *PPP2CA, CDKL3, CDKN2, AIPNL, PHF15, SAR1B, SEC24A, CAMLG, DDX46, C5orf24, TXNDC15, H2AFY, TCF7* | *TRPC7, SMAD5, SPOCK1* | Type II muscle fiber and body weight | - | - |
| 6 | 137.17 – 137.86 | DIAS0000949 - DRGA0006954 | 0.96 | 0.443 | *PGM1, EFCAB7, SNORA16, ALG6, ATG4C* | *CACHD1, UBE2U, ROR1, U6, PGM1* | *ATG4C, DOCK7, ANGPTL3, KANK4, U6, NFIA* | Feed intake and body weight | - | - |
| 1 | 167.09 – 167.88 | MARC0096493 - MARC0054709 | 0.76 | 0.161 | *miRNA, snRNA, CBLN2* | *NETO1* | *No annotated genes* | Average daily gain, feed intake and body weight | ASGA0101718 (167.20), ASGA0106369 (167.28), CASI0008404 (167.76) | - |
| 14 | 31.03 – 31.98 | INRA0043392 - ALGA0076686 | 0.66 | 0.341 | *GTF2H3, EIF2B1, DDX55,TMED2, RILPL1, SNRNP35, RILPL2, SETD8, SBNO1, CDK2AP1, C12ORF65, MPHOSPH9, PITPNM2, ARL6IP4, OGFOD2, ABCB9, VPS37B, HIP1R, CCDC62, DENR, GPR81, Novel protein - coding, RSRC2, ZCCHC8, SNORA9* | *Pseudogene, SCARB1, NCOR2, FAM101A, ZNF664, CCDC92, DNAH10, ATP6V0A2, TCTN2, GTF2H3, U6* | *MLXIP, DIABLO, CCDC147, BCL7A, WDR66, PSMD9, HPD, SETD1B, SSC.93769, TMEM120B, MORN3, ORAI1, KDM2B, IL31, LRRC43, B3GNT4, CLIP1, VPS33A, DIABLO, LRRC43, U6* | Loin muscle area, daily feed intake and birth weight | MARC0017808 (31.11), ALGA0076660 (31.21), ASGA0062485 (31.22), H3GA0039634 (31.29), MARC0041976 (31.48), MARC0094668 (31.67), DIAS0000600 (31.69), ASGA0062507 (31.79) | Haplotype 1: ASGA0062507 (G) - DRGA0013769 (A) - MARC0035603 (A) - INRA0043413 (A) - ALGA0076686 (A); Haplotype 2: INRA0043392 (G) - H3GA0039628 (A) - MARC0017808 (A) - ASGA0062483 (T) - INRA0043359 (T) - ALGA0076659 (T) - ALGA0076660 (G) - ASGA0062485 (C) - H3GA0039634 (A) - ASGA0062487 (G) - ASGA0062490 (G) - ALGA0076663 (G) - ALGA0076667 (A) - H3GA0039636 (G); Haplotype 3: ASGA0062496 (G) - MARC0041976 (T) |
| 1 | 274.12 – 274.98 | INRA0003953 - ALGA0008932 | 0.65 | 0.402 | *Novel protein coding* | *CYLC2* | *COR13C13, OR13C2, OR13F1, OR13C8, OR13C4, OR13F1, ABCA1, NIPSNAP3A, NIPSNAP3A, Olfr270, LOC100739528, COR13C13, LOC100154900, LOC100156114* | Average daily gain, body weight | - | - |
| 7 | 125.00 – 125.98 | M1GA0010987 - ASGA0036842 | 0.65 | 0.368 | *VRK1* | *GLRX5, TCL1B, TCL1A, BDKRB2, Atg2b, BDKRB1, C14orf129, AK7, PAPOLA* | MiRNA | Body weight | - | - |
| 5 | 98.02 – 98.98 | ASGA0103337 - ASGA0026904 | 0.63 | 0.310 | *KITLG, KITL* | *ATP2B1, POC1B* | *TMTC3, CEP290, C12orf29, C12orf50, U6atac* | Meat quality | - | - |
| 17 | 27.03 – 27.85 | ALGA0093855 - ALGA0093900 | 0.52 | 0.370 | *Novel protein coding* | *U11* | *KIF16B, OTOR* | Back fat and body weight | ASGA0075832 (27.29), ALGA0093887 (27.32), ALGA0093891 (27.37), ALGA0093896 (27.47), ALGA0093899 (27.60), H3GA0048148 (27.79) | - |
| 6 | 21.01 – 21.98 | ALGA0034832 - INRA0021283 | 0.51 | 0.301 | *gene - biotype* | No annotated genes | No annotated genes | Average daily gain and body weight | - | - |
| 12 | 41.04 – 41.55 | H3GA0055128 - MARC0051533 | 0.51 | 0.251 | *CCL4, RPL12* | *HEATR6, CCL4, LHX1, AATF, ACACA, C17orf78, TADA2A, DUSP14, SYNRG, DDX52, HNF1B, U6* | *FNDC8, RAD51D, RFFL, LIG3, CCT6B, ZNF830, TMEM132E, CCL11, CCL8, CCL1, CCL2, SNORA70, U6* | Average daily gain and body weight | - |  |
| 16 | 59.13 – 59.98 | H3GA0046720 - H3GA0046791 | 0.49 | 0.353 | *5S - rRNA, SLIT3, MIR218B, protein - coding, U6, PANK3, ssc-mir-103, snRNA, RARS* | *DOCK2, FOXI1, FAM196B, CCDC99* | *ODZ2* | Daily feed intake, body weight and feed conversion ratio | - | - |
| 1 | 172.01 – 172.961 | MARC0076018 - ALGA0116832 | 0.48 | 0.163 | *U6, CDH19* | *5s-RNA* | *CDH19, CDH7* | Average daily gain, feed intake and body weight | MARC0076018 (172.01), ALGA0006516 (172.05), ALGA0006518 (172.10), DRGA0001578 (172.12), ALGA0006519 (172.12), SIRI0000619 (172.23), CASI0009467 (172.24), MARC0051329 (172.42), MARC0004113 (172.88), DRGA0001584 (172.92) | - |
| 1 | 87.01 – 87.85 | DRGA0001180 - H3GA0002131 | 0.43 | 0.340 | *TRAF3IP2, PKC, snRNA, WISP3, TUBE1, C6ORF225, LAMA4, protein - coding* | *Retrotransposed, SLC22A16, CDK19, RPF2, GTF3C6, AMD1, REV3L, KIAA1919, REV3L* | *RFPL4B, NPM1* | Average daily gain, feed intake and body weight | - | - |
| 14 | 29.00 – 29.98 | MARC0020707 - H3GA0039591 | 0.43 | 0.313 | *miRNA, TMEM132B, AACS, BRI3BP, DHX37, UBC, SCARB1* | *TMEM132C* | *SCARB1, FAM101A, ZNF664, DNAH10, ATP6V0A2, NCOR2, CCDC92* | Daily feed intake and body weight | INRA0043334 (29.32), ASGA0062403 (29.27), ALGA0117351 (29.01), ASGA0062403 (29.27), INRA0043333 (29.29), INRA0043334 (29.32) | ASGA0062403 (A) - INRA0043333 (C) - INRA0043334 (A) - ALGA0076573 (C) |
| 17 | 26.04 – 26.99 | INRA0053116 - MARC0053970 | 0.42 | 0.263 | snRNA | *ESF1, NDUFAF5, SEL1L2, MACROD2, FLRT3, U6* | *MACROD2* | Back fat and body weight | - | - |
| 14 | 107.05 – 107.99 | ASGA0065520 - ALGA0080315 | 0.39 | 0.334 | *snoRNA, protein - coding, A1CF, ASAH2, SGMS1* | *DKK1, PRKG1, CSTF2T* | *SGMS1, MINPP1, PAPSS2, ATAD1, PTEN, U6atac* | Average daily gain, daily feed intake and body weight | - | - |
| 4 | 34.06 – 34.96 | ALGA0024499 - DRGA0004712 | 0.37 | 0.320 | *ZFPM2, miRNA* | *ABRA, 7SK, OXR1* | *LRP12, DPYS, DCSTAMP, RIMS2, LOC100354806* | Average daily gain, body weight | - | - |
| 7 | 93.00 – 93.88 | ALGA0043246 - ASGA0034848 | 0.35 | 0.292 | *SLCO3A1, SV2B, pseudogene* | *FAM174B, ST8SIA2, SLCO3A1* | *AKAP13, KLHL25, MTHFD1, SNORA22* | Average daily gain and body weight | H3GA0022261 (93.41) | - |
| 5 | 95.00 – 95.97 | M1GA0008118 - H3GA0017127 | 0.30 | 0.226 | *BTG1, 5S - rRNA* | *EEA1-201, U6, miRNA* | *DCN, LUM, KERA, EPYC, CCER1, U6* | Meat quality | - | - |
| 9 | 24.01 – 24.98 | ASGA0042077 - ALGA0112140 | 0.30 | 0.341 | *CTSC, protein - coding* | *RAB38, TMEM135* | *LOC100524034, NAALAD2, CH242-134A11, TYR, NOX4, LOC100737224, LOC100524034, LOC100524209, LOC100623257, TRIM43, FOLH1* | Average daily gain | - | - |
| 14 | 97.01 – 97.99 | ASGA0065116 - MARC0005727 | 0.30 | 0.289 | *miRNA, ARHGAP22, WDFY4, LRRC18, VSTM4, pseudogene, FAM170B, C10ORF128, snRNA, C10ORF71, DRGX, ERCC6;* | *ZNF488, RBP3, GDF2, GDF10, PTPN20B, FRMPD2, MAPK8, ARHGAP22, MAPK8, U6* | *CHAT, C10orf53, OGDHL, PARG, TIMM23, Psuedogene, NCOA4, MSMB, FAM21A, ZFAND4, MARCH8, ALOX5* | Daily feed intake QTL, body weight, average daily gain | MARC0048620 (97.54), H3GA0041458 (97.83) |  |
| 4 | 36.00 – 36.90 | ALGA0024505 - DIAS0000096 | 0.28 | 0.239 | *protein - coding, DCAF13, SLC25A32, CTHRC1, FZD6, BAALC, ATP6V1C1* | *LRP12, DPYS, DCSTAMP, RIMS2* | *CU633679, NCALD, ATP6V1C1, AZIN1, CU459197, KLF10, CU459197, UBR5, RRM2B, U6* | Average daily gain, body weight | - | - |
| 13 | 36.01 – 36.96 | H3GA0036168 - H3GA0036210 | 0.28 | 0.348 | *TMEM115, CACNA2D2, C3ORF18, HEMK1, miRNA, MAPKAPK3, CISH, DOCK3, SNORD22, RBM15B, MANF, VPRBP* | ZMYND10, NPRL2, CYB561D2, TMEM115, APEH,MST1, RNF123, AMIGO3,GMPPB, IP6K1, CDHR4, FAM212A,CH242-222N14, TRAIP, CAMKV, ACTBL, MST1R, MON1A, RBM6, RBM5, SEMA3F,GNAT1, SLC38A3, SSC.841, SEMA3B,LOC100511718, CCDC36, USP4, IFRD2, RHOA, BSN, C3orf45, HYAL3, NAT6, HYAL1, HYAL2, TUSC2, RASSF1, U6 | RAD54L2, FABP3, TEX264, RAD54L2, GRM2, IQCF6, IQCF3, IQCF5, IQCF2, IQCF1, RRP9, PARP3, GPR62, PCBP4, ABHD14B, ABHD14A, ACY1, RPL29, DUSP7, TWF2, POC1A, GLYCTK, WDR82, PPM1M, TWF2, TLR9, ALAS1, DNAH1, BAP1, PHF7, SEMA3G, TNNC1, NISCH, STAB1, NT5DC2, U6, ssc-mir-135-1, Metazoa_SRP, ssc-let-7g | Average daily gain | - | - |
| 10 | 49.04 – 49.99 | DRGA0010532 - ASGA0048021 | 0.27 | 0.240 | *SLC39A12, 5S - rRNA, CACNB2, NSUN6, RSU1, SNORA72* | *VIM, TMEM236, CH242-21O2, SLC39A12, ST8SIA6* | *C1QL3, PTER, FAM188A, ITGA8, SNORA31, U6* | Average daily gain and body weight | - | - |
| 6 | 50.00 – 50.92 | H3GA0056470 - H3GA0056609 | 0.26 | 0.251 | *PPFIA3, LIN7B, SNRNP70, KCNA7, protein - coding, NTF4, LHB, RUVBL2, GYS1, FTL, BAX-ALPHA, DHDH, NUCB1, TULP2, CCDC155, PTH2, SLC17A7, PIH1D1, ALDH16A1, FLT3LG, RPL13A, SNORD33, snoRNA, SNORD35, RPS11, miRNA, FCRN, RCN3, NOSIP, PRRG2, RRAS, SCAF1, IRF3, BCL2L12, PRMT1, ADM5, CPT1C, TSKS, AP2A1, FUZ, MED25, PTOV1, PNKP, AKT1S1, TBC1D17, IL4I1, NUP62, ATF5, VRK3, ZNF473, snRNA, IZUMO2, pseudogene* | *CRX, SULT2A1, BSPH1, ELSPBP1, CABP5, SPHK2, LIG1, DBP, CA11, NTN5, FUT2A, MAMSTR, RASIP1, C19orf68, ZNF114, CCDC114, IZUMO1, EMP3, TMEM143, LMTK3,FUT1, SSC.75317, BCAT2, CYTH2, KCNJ14, GRWD1, GRIN2D, KDELR1, HSD17B14, SYNGR4, RPL18, PLEKHA4, PPP1R15A, TRPM4, HRC, PPFIA3, CA11, SULT2B1, FAM83E, SPACA4* | *CH242-204P3, MYH14, KCNC3, NAPSA, NR1H2, POLD1, MYBPC2, FAM71E1, EMC10, JOSD2, ASPDH, LRRC4B, SYT3, SHANK1, CLEC11A, GPR32, ACPT, KLK1, KLK15, SSC.11, CD33, IGLON5, VSIG10L, ETFB, CLDND2, NKG7, LIM2, CH242-204P3, ZNF175, LOC100514465, CTU1, SIGLEC5, LOC100516444, HAS1, ZNF726, SNORD88, SNORA19, ssc-mir-125a, ssc-mir-99b, ssc-let-7e* | Average daily gain and body weight | - | - |
| 13 | 23.02 – 23.97 | ALGA0102461 - H3GA0053903 | 0.26 | 0.248 | *STAC, MLH1, LRRFIP2, EPM2AIP1, TRANK1, retrotransposed, pseudogene, protein - coding, DCLK3* | *ARPP21, ssc-mir-128-2, U6* | *CTDSPL, DCLK3, GOLGA4, ITGA9, DLEC1, PLCD1, VILL, MiRNA* | Average daily gain | - | - |
| 14 | 28.02 – 28.97 | ALGA0076463 - M1GA0018457 | 0.26 | 0.257 | TMEM132C | *GLT1D1, SLC15A4, U6, TMEM132C* | *TMEM132B, AACS, BRI3BP, DHX37, UBC, miRNA* | Daily feed intake and body weight | ALGA0076523 (28.45), ALGA0076532 (28.61), MARC0032933 (28.65) | - |
| 4 | 37.05 – 37.99 | H3GA0054755 - ASGA0019336 | 0.25 | 0.263 | *AZIN1, U6, ODF1, KLF10, protein - coding, RRM2B, NCALD* | *RIMS2, DCAF13, SLC25A32, RPL35A, CTHRC1, FZD6, BAALC, ATP6V1C1* | *NCALD, GRHL2, ZNF706, YWHAZ, snoU13, U6* | Average daily gain, body weight | - | - |
| 10 | 15.13 - 15.99 | ALGA0057279 - H3GA0029351 | 0.25 | 0.249 | *WDR26, CNIH3, U3, protein - coding, MIXL1, ACBD3, H3F3A, C1ORF55, LEFTY2, snRNA, PYCR2, TMEM63A;* | *RGS7, FH, KMO, ZNF596, NVL, DEGS1, CNIH4, U6* | *TMEM63A, EPHX1, SRP9, ENAH, LIN9, PARP1, C1orf95, ITPKB, PSEN2* | Average daily gain and body weight | - | - |
| 4 | 33.01 – 33.95 | MARC0016020 - ALGA0024390 | 0.24 | 0.239 | *ABRA, OXR1* | *ANGPT1, U6* | *ZFPM2, MiRNA* | Average daily gain and body weight | - | - |
| 4 | 35.00 – 35.89 | ASGA0019203 - H3GA0012497 | 0.24 | 0.243 | *LRP12, DPYS, TM7SF4, RIMS2* | *ZFPM2, MiRNA* | *RIMS2, DCAF13, SLC25A32, RPL35A, CTHRC1, FZD6, BAALC, ATP6V1C1* | Average daily gain and body weight | - | - |
| 1 | 173.01 – 173.74 | ALGA0006529 - ALGA0006547 | 0.23 | 0.094 | *CDH7* | *CDH19, U6* | *CDH7* | Average daily gain, body weight and feed intake | - | - |
| 6 | 135.07 – 135.95 | INRA0022506 - H3GA0056466 | 0.23 | 0.236 | *LEPOT, DNAJC6, AK3L1, JAK1, ssc-mir-101a-2, 5S_rRNA* | *SERBP1, IL12RB2, IL23RA, SLC35D1, C1orf141, MIER1, PDE4B* | *RAVER2, CACHD1, UBE2U, ROR1, U6* | Average daily gain and body weight | - | - |
| 13 | 21.03 – 21.99 | DRGA0012164 - ALGA0068741 | 0.23 | 0.279 | *CLASP2, PDCD6IP* | *OSBPL10, CMTM6, DYNC1LI1, CMTM7, CMTM8, GPD1L, UBP1, CLASP2* | *ARPP21, ssc-mir-128-2, U6* | Average daily gain | - | - |
| 14 | 43.01 – 43.99 | ASGA0062929 - ASGA0062986 | 0.23 | 0.209 | *CABP1, MLEC, UNC119B, ACADS, SPPL3,HNF1A, C12ORF43, OASL,ANKRD13A, TCHP, GLTP, TRPV4, C12ORF34, MVK, MMAB, UBE3B, KCTD10, MYO1H* | *Pseudogene, HECTD4, TRAFD1, NAA25, POP5, ERP29, TMEM116, ADAM1B, MAPKAPK5, ALDH2, ADAM1A, CCDC64, PLA2G1B, RAB35, GCN1L1, MSI1, SIRT4, COX6A1, TRIAP1, GATC, SRSF9, Rnf10, COQ5, DYNLL1,* U6, U4 | SART3, FICD, CMLKR1, MYO1H, FOXN4, ACACB, UNG, ALKBH2, USP30, SVOP, DAO, SSH1, CORO1C, SELPLG, LOC100621401, ISCU, U1 | Daily feed intake and body weight | - | - |
| 14 | 126.01 – 126.98 | ALGA0081288 - ALGA0081327 | 0.23 | 0.209 | *SORCS3* | *ITPRIP, CCDC147, WDR96, GSTO1* | *SORCS1, U6* | Average daily gain, feed intake and body weight | - | - |
| 4 | 88.06 – 88.94 | INRA0015178 - ASGA0020484 | 0.22 | 0.196 | *GORAB, METTL11B, KIFAP3, SCYL3, METTL18, C1OEF112, SELL* | *KIAA0146, CEBPD, MCM4, HENMT1, PRRX1, DNAPK* | *SELL, SELP, F5, SSC.22020, C1orf114, BLZF1, NME7, ATP1B1* | Average daily gain and body weight | - | - |
| 9 | 27.04 – 27.98 | ALGA0106031 - ASGA0101939 | 0.22 | 0.215 | *No annotated genes* | *CHORDC1* | *FAT3, eef1a1l2, MTNR1B, SLC36A4* | Average daily gain | - | - |
| 1 | 113.06 – 113.93 | CASI0004348 - MARC0022681 | 0.21 | 0.199 | *No annotated genes* | *No annotated genes* | *MBD2, POLI, STARD6, C18orf54, C18orf26, RAB27B, CCDC68* | Average daily gain, feed intake and body weight | - | - |
| 15 | 135.05 – 135.99 | ASGA0070932 - ALGA0087385 | 0.21 | 0.285 | *U6, SNORA31* | *ASIC4, GMPPA, NHEJ1, GLB1L, SLC23A3, CNPPD1, FAM134A, ZFAND2B, ABCB6, ATG9A, ANKZF1, STK16, TUBA4A, CCDC108, IHH, DNAJB2, PTPRN, RESP18,DNPEP,INHA,OBSL1,TMEM198, DES, CHPF,ssc-mir-4334* | *EPHA4* | Average daily gain, feed intake and body weight | - | - |
| 1 | 175.11 – 175.97 | MARC0017765 - ALGA0006569 | 0.20 | 0.119 | *SERPINB 2, SERPINB 5, SERPINB 7, SERPINB 8, SERPINB 10, BCL2, KDSR, VPS4B* | *No annotated genes* | *PHLPP1, ZCCHC2, TNFRSF11A, KIAA1468, PIGN, MiRNA, U6* | Average daily gain, feed intake and body weight | - | H3GA0003077 (A) - INRA0004847 (G) - MARC0035436 (C) - INRA0004853 (T) - H3GA0003080 (G) - ASGA0004958 (T) - INRA0004857 (A) |

^@^ The 1Mb windows are presented in descending order based on the percent genetic variance explained greater than 0.2%.

*Posterior probability that the SNPs in 1Mb window could explain the genetic variance greater than zero (PPA: Posterior probability of association).

**Association of single SNPs was considered based on genomic control corrected P-values at a threshold of 0.01 by the PLINK software

***Association of haplotypes was considered based on genomic control corrected P-values at a threshold of 0.05 by the PLINK software

^$^ The genes and their abbreviations are based on *Sus scrofa* genome build 10.2

Note: The windows with unmapped SNPs are not real consecutive SNP windows and hence they are not presented
