# Supplementary material for: Whole Genome Association Studies of Residual Feed Intake and Related Traits in the Pig
Source: PLoS One. 2013 Jun 26;8(6):e61756. doi: 10.1371/journal.pone.0061756 (PMC3694077; doi:10.1371/journal.pone.0061756)
Supplement: Table S5 — Detailed information about candidate QTL regions associated with the back fat (BF) by 1 Mb SNP window, single SNP and haplotype analyses. (DOCX) [file pone.0061756.s007.docx]

**Table S5. Detailed information about candidate QTL regions associated with the back fat (BF) by 1Mb SNP window, single SNP and haplotype analyses.**

| SSC | Location (start-end) in Mb^@^ | 1Mb SNP window | Percent genetic variance explained | PPA *(P > 0) | Genes within the SNP window^$^ | Genes within 1Mb upstream the SNP window^$^ | Genes within 1Mb downstream the SNP window^$^ | Previously reported important QTL at the SNP window | Associated single SNPs (position in Mb) within the 1Mb window** | Associated haplotypes within the 1Mb window*** |
| --- | --- | --- | --- | --- | --- | --- | --- | --- | --- | --- |
| 7 | 112.05-112.96 | ALGA0044374-DRGA0008090 | 1.16 | 0.632 | *Env protein* | *SNORA18, MiRNA* | *7SK* | Average daily gain, body weight, back fat and loin muscle area | - | - |
| 3 | 47.04-47.98 | MARC0085867-ALGA0018683 | 0.86 | 0.499 | *protein_coding, SEPT10, NPHP1, ACOXL, BCL2L11, BUB1-like* | *FBLN7, MERTK, ANAPC1, Gm355, SNORA70, MiRNA* | *KCNIP3, FAHD2A, GPAT2, ADRA2B, TPC3, ARID5A, LOC100524797, NPHP1, MALL, MRPS5, ZNF514, MAL, ZNF2, , PROM2, MiRNA, U6* | Body weight, daily gain, feed conversion ratio and loin muscle area | - | - |
| 18 | 55.03-55.96 | ALGA0119800-ALGA0123577 | 0.83 | 0.550 | *Pseudogene, RAMP3, WAP, TBRG4, SNORA21, SNORA5, snoRNA, protein_coding, CCM2, PPIA, H2A.Z, miRNA, MYO1G, SNORA9, OGDH, TMED4, DDX56, NPC1L1, NUDCD3, CAMK2B, YKT6, GCK, MYL7, POLD2, AEBP1* | *CH242-79N16, CH242-226A14, CH242-64G23, LOC617503, C1GALT1C1, MCTS1, CUL4B, ATP1B4, LAMP2, FAM70A* | *CH242-445E7, CH242-41K13, AKAP17A, CT47B1, LONRF3, ZCCHC12, IL13RA1, DOCK11, 5S_rRNA, MiRNA, U6* | Intra muscular fat | - | - |
| 16 | 48.02-48.99 | ALGA0090558-ASGA0073294 | 0.71 | 0.48 | *No genes* | *ADAMTS6, CENPK, PPWD1, TRIM23, C16H5orf44, SGTB, NLN, RPL36A, ERBB2IP* | *MAST4, CD180* | Body weight, daily feed intake, feed conversion ratio and back fat | - | - |
| 18 | 43.01-43.94 | ASGA0079708-ALGA0098147 | 0.68 | 0.424 | *BMPER, miRNA, BBS9* | *7-SEP, HERPUD2, TBX20, DPY19L2, NPSR1, U6* | *BBS9, PTHB1-like, LOC100738822, NT5C3, FKBP9, RP9, KBTBD2, HMGN1, LSM5, AVL9* | Body weight and feed conversion ratio and back fat | - | - |
| 2 | 125.01-125.99 | ALGA0117661-ALGA0015536 | 0.64 | 0.408 | *protein_coding,C11ORF46,COMMD10, SEMA6A* | *TRIM36, PGGT1B, CCDC112, TICAM2, FEM1C, TMED7, CDO1, ATG12* | *No annotated genes* | Body weight, daily gain, feed intake, back fat and loin muscle area | ALGA0107733 (125.74) | - |
| 19 | 113.08-113.96 | ALGA0099955-ASGA0105482 | 0.55 | 0.484 | *FAM70A, ZBTB33, RHOXF1, protein_coding, NKAP, AKAP14, NDUFA1, RNF113A, UPF3B; SNORA69, ANKRD58, SEPT6, miRNA, NKRF, UBE2A, CXORF56, SLC25A6, SLC25A43, PGRMC1, AKAP17A* | *CH242-79N16, CH242-226A14, CH242-226A14, CH242-64G23, LOC617503, C1GALT1C1, MCTS1, CUL4B, ATP1B4, LAMP2, FAM70A* | *CH242-445E7, CH242-41K13, AKAP17A, CT47B1, LONRF3, ZCCHC12, IL13RA1, DOCK11, 5S_rRNA, MiRNA, U6* | Back fat | - | - |
| 13 | 34.12-34.99 | MARC0078942-ASGA0057115 | 0.50 | 0.427 | *NPG3, NPG5, protein_coding, PMAP23, pseudogene, PLXNA1, NME6, CAMP, CCDC51, CCDC72, ATRIP, PFKFB4, COL7A1, miRNA, UQCRC1, SHISA5, TREX1, TMEM89, SLC26A6, CELSR3, NCKIPSD, U6, PRKAR2A, SLC25A20, ARIH2, P4HTM, WDR6, DALRD3, MIR425, MIR191, NDUFAF3, IMPDH2, QRICH1, USP19, LAMB2,* | *NBEAL2, NRADD, SETD2, NBEAL2, KLHL18, SCAP, Pseudogene, ELP6, CSPG5, SMARCC1, DHX30, MAP4, LOC638399, MTAP4* | *APEH, MST1, RNF123, AMIGO3, GMPPB, IP6K1, CDHR4, FAM212A, CH242-222N14, TRAIP, CAMKV, LOC100626209, MST1R, MON1A, RBM6, RBM5, SEMA3F, GNAT1, SLC38A3, SSC.841,CCDC71, KLHDC8B, SEMA3B, LOC100511718, CCDC36, USP4, IFRD2, RHOA, BSN, C3orf45, HYAL3, NAT6, HYAL1, HYAL2, TUSC2, U6* | *Average daily gain and back fat area* | - | - |
| 3 | 119.01-119.98 | ASGA0016106-ALGA0021085 | 0.44 | 0.459 | *CGREF1, KHK, EMILIN1, AGBL5, TMEM214, MAPRE3, DPYSL5, CENPA,C2ORF18, pseudogene, CIB4, C2ORF70, EPT1, GPR113, LCTHIO, HADHA,CCDC164, FAM59B, RAB10, U6, KIF3C* | *CGREF1, BRE, RBKS, SLC4A1AP, SUPT7L, GPN1, CCDC121, ZNF512, C2orf16, EIF2B4, SNX17, PPM1G, NRBP1, IFT172, KRTCAP3, FNDC4, GCKR, ATRAID, SLC5A6, TCF23, C2orf53, PREB, ABHD1* | *KIF3C, ASXL2, DTNB, DNMT3A, POMC, EFR3B* | Body weight, average daily gain and back fat | - | - |
| 6 | 152.10-152.98 | CASI0007691-ASGA0030228 | 0.43 | 0.395 | *protein_coding, CYP4A24, CYP4A21, CYP4B1, KIAA0494, ATPAF1-AS1, SNORA73, miRNA, snRNA, MOB3C, MKNK1, DMBX1, FAAH, NSUN4, UQCRH, LRRC41, RAD54L, LURAP1, NP_001072137.1, TSPAN1, PIK3R3, MAST2* | *FOXE3, CMPK1, STIL, TAL1, CYP4A24, CYP4X1* | *PRDX1, MMACHC, TESK2, TOE1, MUTYH, HPDL, ZSWIM5, EIF2B3, UROD, HECTD3, U6* | - | - | - |
| 2 | 28.01-28.98 | ALGA0124274-INRA0059415 | 0.42 | 0.401 | *SLC1A2, CD44, PDHX, miRNA, APIP, EHF, ELF5, CAT, ABTB2* | *FJX1, PAMR1, COMMD9, LDLRAD3, TRIM44* | *LMO2, KIAA1549L, C11orf91, CD59, FBXO3, ABTB2, NAT10, CAPRIN1, SNORA18* | Body weight, daily gain, back fat and loin muscle area | - | - |
| 14 | 122.04-122.97 | ASGA0066137-MARC0075113 | 0.42 | 0.401 | *BTRC, POLL, DPCD, FBXW4, protein_coding, FGF8, NPM3, MGEA5, KCNIP2, C10ORF76, HPS6, NP_001072153.1* | *HIF1AN, PAX2, SEMA4G, PDZD7, KAZALD1, LZTS2, C10orf2, PDZD7, SFXN3, PDZD7, MRPL43, TLX1, LBX1, U6, 7SK* | *PITX3, GBF1, NFKB2, NFKB2, PSD, TMEM180, ACTR1A, SUFU, TRIM8, ARL3, SFXN2, ARL3, SFXN2, WBP1L, CYP17A1, C10orf32, AS3MT, CNNM2, LDB1, PPRC1, NOLC1, ELOVL3, ssc-mir-146b* | Daily feed intake, body weight, average daily gain, loin muscle area and back fat | - | - |
| 8 | 119.15-119.96 | DIAS0001763-ALGA0049192 | 0.41 | 0.32 | *PITX2C* | *ALPK1, TIFA, AP1AR, SNORA76* | *PLA2G12A, CASP6, CCDC109B, SEC24B, No protein product, ENPEP, ELOVL6, EGF, LRIT3, RRH, GAR1, CFI, MiRNA* | loin muscle area and back fat | CASI0008396 (119.30), DIAS0003532 (119.16) | - |
| 7 | 119.02-119.95 | MARC0011341-ALGA0045055 | 0.36 | 0.386 | *TTC7B, protein_coding, C14ORF159, GPR68, CCDC88C, SMEK1, CATSPERB, snRNA, TC2N* | *EFCAB11, TDP1, KCNK13, PSMC1, C14orf102, CALM1, TTC7B* | *GOLGA5, TC2N, FBLN5, TRIP11, ATXN3, CPSF2, NDUFB1, SLC24A4* | Average daily gain, body weight, back fat and loin muscle area | - | - |
| 5 | 68.01-69.00 | ASGA0025952-M1GA0007884 | 0.35 | 0.316 | *AKAP3, DYRK4, RAD51AP1, C12ORF4, FGF6, FGF23, C12ORF5, CCND2, PARP11, PRMT8* | *VWF, ANO2, NTF3, KV1.5, KCNA1, KCNA6, GALNT8, NDUFA9, AKAP3* | *IQSEC3, SLC6A12, SLC6A13, CCDC77, KDM5A, Pseudogene, PRMT8, EFCAB4B, TSPAN11, TSPAN9, TEAD4, TULP3, RHNO1, FOXM1, NRIP2, ITFG2, FKBP4, DDX11, WASH1* | Average daily gain and back fat | H3GA0016585 (68.36) | H3GA0016585 (T) - MARC0051602 (G) |
| 17 | 26.05-26.99 | INRA0053116-MARC0053970 | 0.33 | 0.296 | *snRNA* | *ESF1, NDUFAF5, SEL1L2, MACROD2, FLRT3, U6* | *MACROD2* | Back fat | - | - |
| 19 | 37.03-37.99 | MARC0025293-ASGA0081002 | 0.32 | 0.319 | *Pseudogene,*  *XK, CYBB, DYNLT3, CXORF27, protein_coding, SYTL5, SRPX, U6* | *CH242-280I8, LOC100520991, FTH1, FAM47A, PRRG1* | *CH242-137L16, CH242-81B3, MID1IP1, TSPAN7, Fgd1, RPGR, OTC* | Average daily gain, back fat | - | - |
| 11 | 8.01-8.93 | INRA0034855-ASGA0049660 | 0.31 | 0.36 | *RXFP2* | *WDR95, HSPH1, B3GALTL, HMGB1, USPL1, ALOX5AP, C13orf33, TEX26, U6, MiRNA* | *PDS5B, KL, STARD13, U6* | Back fat and loin muscle area | - | - |
| 17 | 57.04-57.94 | ASGA0077474-ASGA0077537 | 0.31 | 0.352 | *ARFGEF2, CSE1L, protein_coding, STAU1, KCNB1, PTGIS, pseudogene, B4GALT5, SLC9A8, TMEM189,UBE2V1* | *PREX1, ARFGEF2* | *CH242-7P5, CEBPB, PTPN1, FAM65C, PARD6B, BCAS4, ADNP, DPM1, MOCS3, KCNG1* | Back fat | - | - |
| 14 | 29.00-29.99 | MARC0020707-H3GA0039591 | 0.30 | 0.349 | *miRNA, TMEM132B, AACS, BRI3BP, DHX37, UBC* | *TMEM132C* | *Psuedogene, SCARB1, NCOR2, FAM101A, ZNF664, CCDC92, DNAH10, ATP6V0A2, U6* | Daily feed intake, body weight, back fat and loin muscle area | - | - |
| 18 | 42.03-42.94 | ASGA0079687-INRA0055780 | 0.29 | 0.281 | *SEPT7, protein_coding, TBX20, DPY19L2, DPY19L1, NPSR1, U6* | *KIAA0895, ATP6V0A4, 7-Sep, AOAH, ANLN, U6* | *BMPER* | Body weight, feed conversion ratio and back fat | - | - |
| 6 | 126.02-126.99 | ASGA0029527-MARC0076427 | 0.28 | 0.284 | *AK5, protein_coding, misc_RNA* | *SURF6, GIPC2, DNAJB4, FUBP1, NEXN, FAM73A, USP33, ZZZ3, AK5, U6* | *ST6GALNAC3, MSH4, RABGGTB, ACADM, SLC44A5, SNORD45, U1, U6* |  | - | - |
| 19 | 13.09-13.99 | ASGA0080840-ASGA0091237 | 0.27 | 0.258 | *ACE2, CA5B, protein_coding, AP1S2, GRPR, POLR3K, CTPS2;* | *CH242-100L3, CH242-168N3, PIGA, FIGF, PIR, BMX, ACE2, GLRA2, FANCB, MOSPD2, ASB9, ASB11* | *CTPS2, S100G, SYAP1, TXLNG, RBBP7, REPS2, NHS* | Feed intake, daily gain, back fat | - | - |
| 19 | 143.01-143.5 | M1GA0023917-CAHM0000013 | 0.27 | 0.326 | *BRCC3, MTCP1NB, FUNDC2, F8, protein_coding, miRNA, MPP1, DKC1, SNORA36, GAB3, snRNA, IKBKG, G6PD* | *PDZD4, L1CAM, CH242-139K22.10, CH242-305A15.4, AVPR2, ARHGAP4, SLC6A8, BCAP31, ABCD1, CH242-139K22.7, NAA10, PLXNB3, SRPK3, IDH3G, RENBP, HCFC1, TMEM187, IRAK1, MECP2, OR6C2, Olfr800, SSR4, LOC100153523, Olfr790, SPRY3, TMLHE, CLIC2, RAB39B, VBP1, BRCC3, SNORA42, MiRNA, U6* | *G6PD, FAM3A, SLC10A3, UBL4A, LAGE3, PLXNA3, FAM50A, GDI1, MXRA5, PRKX, U6* | Average back fat thickness | - | - |
| 10 | 30.01-30.98 | ASGA0047227-ALGA0116145 | 0.25 | 0.314 | *protein_coding, HSD17B3, C9orf102,PTCH1, 5S_rRNA* | *SYT2, KDM5B, LOC516589, RABIF, KLHL12, ADIPOR1, CYB5R1, KDM5B, LOC516288, HABP4, CDC14B, SLC35D2, ZNF367,SLC35D2, HSD17B3, Pseudogene* | *FANCC, C10H9orf3, FBP1, FBP2, DAPK1, CTSL1, U6, ssc-mir-27b, ssc-mir-23b, ssc-mir-24-2* | Average daily gain and average back fat thickness | - | - |
| 9 | 113.05-113.94 | ASGA0044098-MARC0059476 | 0.24 | 0.220 | *FGL2, CCDC146, FAM185A, PMPCB* | *PHTF2, PTPN12, MAGI2* | *Psuedogene, ORC5, DNAJC2, Psmc2, SLC26A5, ERVI-1, RELN* | back fat | - | - |
| 2 | 19.08-19.85 | ALGA0103993-ALGA0012364 | 0.23 | 0.216 | *CD82, EXT2* | *FUCT1, CHST1, SYT13, PRDM11, TP53I11, TSPAN18* | *EXT2, ACCS, ACCSL, ALKBH3, HSD17B12, TTC17, API5, U6, ssc-mir-129a, MiRNA, U6* | Average back fat thickness and loin muscle area | - | - |
| 5 | 34.02-34.95 | SIRI0000534-ALGA0031642 | 0.23 | 0.289 | *GRIP1* | *LLPH, TMBIM4, IRAK3, HELB, GRIP1, Psuedogene, MSRB3, HMGA2, MiRNA, 5S_rRNA, U6* | *CAND1, IL26, IFNG, IL22, MDM1, RPL31, RAP1B, NUP107, SLC35E3, MDM2* | back fat | - | - |
| 10 | 10.00-11.00 | ALGA0056841-H3GA0029163 | 0.23 | 0.289 | *RRP15, TGFB2* | *GPATCH2, SPATA17, U6ATAC, ATL3, SLC22A10* | *LYPLAL1, MPHOSPH6, SLC30A10, EPRS, BPNT1, IARS2, RAB3GAP2, IARS2, ssc-mir-215, ssc-mir-194b, SNORA36* | No QTL | - | - |
| 14 | 24.04-24.91 | ALGA0076158-MARC0069598 | 0.23 | 0.284 | *CHFR, FBRSL1, GOLGA3, P2RX2, GALNT9, NOC4L, PUS1, ULK1, EP400* | *SPOCK3, ZNF268, ANHX, ZNF84, ZNF26, Olfr790, U6* | *ULK1, MMP17, SFSWAP, GPR133, RAN, STX2* | No QTL | - | - |
| 1 | 249.03-250 | MARC0099224-DIAS0002810 | 0.22 | 0.212 | *APBA1, C9ORF135, PTAR1, MAMDC2, C10orf112* | *TJP2, FAM189A2, PGM5, TMEM252, FAM122A, APBA1, PIP5K1B* | *KLF9, SMC5, TRPM3, ssc-mir-204* | No QTL | - | - |
| 7 | 118.00-118.98 | ALGA0044788-H3GA0023105 | 0.22 | 0.311 | *EFCAB11, TDP1, KCNK13, PSMC1, C14ORF102, CALM1, TTC7B* | *FOXN3, ZC3H14, EML5, TTC8, 7SK, U6, ATP6V1G1* | *TTC7B, RPS6KA5, C14orf159, GPR68, CCDC88C, SMEK1, CATSPERB, TC2N, U6ATAC* | No QTL | - | - |
| 6 | 50.01-50.92 | H3GA0056470-H3GA0056609 | 0.21 | 0.251 | *SNORD33,SNORD35, NUP62, PPFIA3, LIN7B, SNRNP70, KCNA7, NTF4, LHB, RUVBL2, GYS1, FTL, BAX-ALPHA, DHDH, NUCB1, TULP2, CCDC155, PTH2, SLC17A7, PIH1D1, ALDH16A1, FLT3LG, RPL13A, RPS11, FCRN, RCN3, NOSIP, PRRG2, RRAS, SCAF1, IRF3, BCL2L12, PRMT1, PTOV1, ADM5, CPT1C, PNKP, TSKS, AP2A1, AKT1S1, TBC1D17, FUZ, MED25, IL4I1, ATF5, VRK3, ZNF473, IZUMO2* | *CRX, SULT2A1, BSPH1, ELSPBP1, CABP5, SPHK2, LIG1, DBP, CA11, NTN5, FUT2A, FUT2, MAMSTR, RASIP1, C19orf68, ZNF114, CCDC114, IZUMO1, EMP3, TMEM143, LMTK3, LMTK3, FUT1, SSC.75317, BCAT2, CYTH2, KCNJ14, GRWD1, GRIN2D, KDELR1, HSD17B14, SYNGR4, RPL18, PLEKHA4, PPP1R15A, TRPM4, HRC,PPFIA3, SPHK2, CA11, SEC1, LMTK3, SULT2B1, FAM83E, SPACA4, RPL18* | *CH242-204P3.6, MYH14, KCNC3, NAPSA, NR1H2, POLD1, MYBPC2, FAM71E1, EMC10, JOSD2, ASPDH, LRRC4B, SYT3, SHANK1, CLEC11A, GPR32, ACPT, KLK1, KLK15, SSC.11, CH242-204P3.7, CD33, IGLON5, VSIG10L, ETFB, CLDND2, NKG7, LIM2, CH242-204P3.4, ZNF175, LOC100514465, CTU1, SIGLEC5, LOC100516444, HAS1, ZNF726, SNORD88, SNORA19, ssc-mir-125a, ssc-mir-99b, ssc-let-7e* | No QTL | - | - |
| 9 | 43.06-43.97 | ALGA0106351-ALGA0052463 | 0.21 | 0.32 | *ARHGAP20, POU2AF1, C11ORF93, C11ORF53* | *RDX, C11orf87, ZC3H12C, U1* | *Sik2, LAYN, C11orf88, BTG4, SIK2, PPP2R1B, ALG9,FDXACB1, C11orf1, CRYAB, LOC100520143, C11orf52, DIXDC1, DLAT, PIH1D2, C11orf57, TIMM8B, SDHD, IL18, TEX12,BCO2, PTS, PLET1, ssc-mir-34c-1* | No QTL | - | - |
| 12 | 27.01-28.00 | ALGA0065768-ALGA0065830 | 0.21 | 0.297 | *CACNA1G, ABCC3, ANKRD40, LUC7L3, WF!KKN2, TOB1, SPAG9, NME2, NME2, MBTD1, UTP18* | *CH242-301O20.6, CH242-301O20.3, TAC4, DLX3, ITGA3, PDK2, SAMD14, PPP1R9B, SGCA, HILS1, COL1A1, TMEM92, LRRC59, EME1, MRPL27, XYLT2, Lrrc37a, LRRC37B, RDM1, ACSF2, CHAD, RSAD1, SPATA20, MYCBPAP, EPN3, CACNA1G, U6* | *CA10* | Back fat | - | - |
| 14 | 28.02-28.97 | ALGA0076463-M1GA0018457 | 0.21 | 0.278 | *TMEM132C* | *GLT1D1, SLC15A4, TMEM132C, U6* | *TMEM132B, AACS, BRI3BP, DHX37, UBC, MiRNA* | Average back fat thickness and loin muscle area | - | - |
| 17 | 11.03-11.95 | ALGA0093209-ASGA0075368 | 0.21 | 0.260 | *ADAM18, ADAM3A, ZMAT4, SFRP1* | *CSGALNACT1, SH2D4A, LOC100737676, IDO1, ADAM18, ADAM3A* | *CHRNB3, C8orf40, SLC20A2, VDAC3, GOLGA7, GINS4, GOLGA7, AGPAT6, ANK1, KAT6A, RNF170, THAP1, CHRNA6, ssc-mir-486-2, 5S_rRNA* | Average back fat thickness | - | - |
| 19 | 104.09-104.96 | ASGA0105262-ASGA0100189 | 0.21 | 0.207 | *RGAG1, CHRDL1* | *COL4A6, COL4A5, AMMECR1, MiRNA, U6* | *LOC100521239, GUCY2F, GAPDH, NXT2, KCNE1L, CH242-17O13.4, ACSL4, CAPN6, DCX* | Average back fat thickness | - | - |
| 7 | 31.01-31.99 | ALGA0039868-ASGA0032245 | 0.20 | 0.275 | *TINAG, LRRC1, KLHL31, GCLC, KHDRBS2* | *HMGCLL1, GFRAL, TMP-HCRTR2, FAM83B, TINAG* | *Psuedogene, KHDRBS2, PRIM2, U6, 7SK* | Average daily gain and body weight | - | - |
| 8 | 120.00-121.00 | ASGA0090428-ALGA0049254 | 0.20 | 0.224 | *ENPEP, ELOVL6, EGF, LRIT3, RRH, GAR1, CFI, PLA2G12A, CASP6, CCDC109B, SEC24B* | *PITX2* | *COL25A1, AGXT2L1, OSTC, RPL34, SNORA18, U11* | Fat to meat ratio | H3GA0025336 (120.73) | Haplotype 1: ALGA0105488 (T) - ALGA0049192 (T) - ASGA0090428 (A) - MARC0030774 (A) - ALGA0049202 (A) - ASGA0091658 (C); Haplotype 2: ALGA0049233 (T) - ASGA0039666 (G) - ASGA0039646 (A) - M1GA0012034 (T) - H3GA0025336 (T) - M1GA0012035 (A) -MARC0025408 (G) - ALGA0049249 (T) - ALGA0049254 (G) |
| 11 | 16.05-17.00 | ASGA0049979-MARC0028819 | 0.20 | 0.265 | *THSD1, CKAP2, NEK3, NEK5, ALG11, CCDC70, INTS6, SERPINE3, FAM124A* | *FOXO1, MRPS31, SLC25A2, TPTE2, VPS36, THSD1, SNORA1* | *DLEU7* | Back fat and loin muscle area | - | - |

^@^ The 1Mb windows are presented in descending order based on the percent genetic variance explained greater than 0.2%.

*Posterior probability that the SNPs in 1Mb window could explain the genetic variance greater than zero (PPA: Posterior probability of association).

**Association of single SNPs was considered based on genomic control corrected P-values at a threshold of 0.01 by the PLINK software

***Association of haplotypes was considered based on genomic control corrected P-values at a threshold of 0.05 by the PLINK software

^$^ The genes and their abbreviations are based on *Sus scrofa* genome build 10.2

Note: The windows with unmapped SNPs are not real consecutive SNP windows and hence they are not presented
