# Supplementary material for: Whole Genome Association Studies of Residual Feed Intake and Related Traits in the Pig
Source: PLoS One. 2013 Jun 26;8(6):e61756. doi: 10.1371/journal.pone.0061756 (PMC3694077; doi:10.1371/journal.pone.0061756)
Supplement: Table S6 — Detailed information about candidate QTL regions associated with the loin muscle area (LMA) by 1 Mb SNP window, single SNP and haplotype analyses. (DOCX) [file pone.0061756.s008.docx]

**Table S6. Detailed information about candidate QTL regions associated with the loin muscle area (LMA) by 1Mb SNP window, single SNP and haplotype analyses.**

| SSC | Location (start-end) in Mb^@^ | 1Mb SNP window | Percent genetic variance explained | PPA (P > 0)* | Genes within the SNP window^$^ | Genes within 1Mb upstream the SNP window^$^ | Genes within 1Mb downstream the SNP window^$^ | Previously reported important QTL at the SNP window | Associated single SNPs (position in Mb) within the 1Mb window** | Associated haplotypes within the 1Mb window*** |
| --- | --- | --- | --- | --- | --- | --- | --- | --- | --- | --- |
| 7 | 31.01 - 31.99 | ALGA0039868 - ASGA0032245 | 6.83 | 0.928 | *Protein - coding; LRRC1; KLHL31; GCLC; pseudogene; KHDRBS2* | *HMGCLL1, GFRAL, TMP, HCRTR2, FAM83B, TINAG* | *Pseudogene, KHDRBS2, PRIM2, U6, 7SK* | Average daily gain and body weight | ALGA0039866 (31.02), ALGA0039930 (31.27), ASGA0032215 (31.60), H3GA0020592 (31.71), ALGA0039974 (31.82), MARC0010879 (31.86), ASGA0032239 (31.87), MARC0098266 (31.94) | Haplotype 1: ALGA0039974 (C) - H3GA0020604 (T) - MARC0010879 (A); Haplotype 2: MARC0053311 (C) - H3GA0020592 (A) - MARC0050857 (T); Haplotype 3: ALGA0039921 (A) - ALGA0039930 (G); Haplotype 4: MARC0053311 (C) - H3GA0020592 (C) - MARC0050857 (T); Haplotype 5: M1GA0009837 (A) - SIRI0001104 (G) - ASGA0032205 (C) - ASGA0085466 (A) - MARC0114641 (T) - ALGA0122542 (C) - ALGA0039947 (T) |
| 17 | 69.06 - 69.33 | ALGA0115285 - M1GA0022883 | 3.28 | 0.865 | *TAF4;PSMA7;LSM14B;protein - coding;RPS21;CABLES2;C20ORF151;GATA5;ssc-mir-133a; ssc-mir-1a;* | *Novel protein, CDH4, TAF4, U6* | *No annotated genes* | Body weight | M1GA0022894 (69.30) | ASGA0078573 (A) - M1GA0022894 (T) - M1GA0022883 (C) |
| 16 | 25.04 – 26.00 | ALGA0121589 - ALGA0089813 | 3.03 | 0.743 | *OSMR;RICTOR;U6;U4;miRNA;FYB;DAB2;C9;* | *WDR70, EGFLAM, CH242-209G14.2, LIFR, MiRNA, U6* | *PRKAA1, U2, 5S_rRNA* | Back fat above loin muscle dorsi | H3GA0046318 (25.60); ALGA0089846 (26.48); H3GA0046337 (26.74) | - |
| 7 | 63.07 - 63.96 | ASGA0034265 - H3GA0021944 | 1.11 | 0.45 | *C15ORF39, miRNA,U6,PPCDC,SCAMP5,RPP25,COX5A, C7H15orf17, MPI, SCAMP2,ULK3, CPLX3, LMAN1L, CSK, CYP1A2, CYP1A1;EDC3;CLK3;ARID3B;UBL7; protein - coding;* | *LINGO1, CSPG4, SNX33, IMP3, SNUPN, PTPN9, SIN3A, MAN2C1, NEIL1, COMMD4, Trcg1, C15orf39, PEAK1, HMG20A, MiRNA, U6* | *ISLR, PML, STOML1, LOXL1, TBC1D21, C15orf59, NPTN, C15orf60, HCN4, NEO1, Pseudogene,STRA6* | Average daily gain, body weight, loin muscle depth, area, loin weight | - | - |
| 9 | 151.01 - 151.94 | M1GA0013442 - MARC0087440 | 0.92 | 0.575 | *U6;protein - coding;* | *IKZF1, FIGNL1, AADC, DDC, GRB10, COBL, U6* | *No annotated genes* | Hemoglobin and carcass weight | ASGA0105343 (151.01), H3GA0028781 (151.39) | - |
| 2 | 65.06 - 65.99 | ASGA0096884 - M1GA0002934 | 0.64 | 0.304 | *CLEC17A;CD97;DDX39A;protein coding;PKN1;PTGER1;GIPC1;DNAJB1;TECR;NDUFB7;LPHN1;ASF1B;PRKACA;C19ORF67;miRNA;PALM3;IL27RA;RLN3;RFX1;DCAF15;PODNL1;CC2D1A;C19ORF57;NANOS3;MIR181D;ssc-mir-181c;ssc-mir-23a;ssc-mir-27a;ssc-mir-24;ZSWIM4;C19ORF53;MRI1;CCDC130;CACNA1A* | *LOC100626190, EMR2, LOC100516420, LOC100518239, LOC100518060, LOC484897, EMR3, ZNF333, SNORA31* | *GADD45GIP1, GCDH, TRMT1, LYL1, NFIX, MAST1, DNASE2, KLF1, SYCE2, BEST2, HOOK2, JUNB, PRDX2, RNASEH2A, ASNA1, CACNA1A, C19orf43, TNPO2, IER2, STX10, FBXW9, GNG12, DHPS, NACC1, WDR83, FARSA, WDR83OS, MAN2B1, CALR, RAD23A, ZNF564, ZNF791, ZNF709, ZNF14, SNORD41, MiRNA, 5S_rRNA* | Average daily gain, body weight and loin muscle depth |  |  |
| 7 | 32.02 - 32.96 | ALGA0039990 - ASGA0032302 | 0.62 | 0.253 | *KHDRBS2;pseudogene;PRIM2;U6* | *Pseudogene, TINAG, MLIP, LRRC1, KLHL31, GCLC, KHDRBS2, 7SK* | *PRIM2, RAB23, BAG2, ZNF451, BEND6,DST, COL9A3, SNORA72, MiRNA* | Average daily gain | - | - |
| 14 | 87.03 - 87.98 | H3GA0041116 - MARC0113429 | 0.57 | 0.26 | *No annotated genes* | *DLG5, POLR3A, RPS24* | *ZMIZ1, CypD, ANXA11, PLAC9, C10orf57, SFTPD, CH242-504L17.3, SFTPA1, MAT1A, DYDC1, FAM213A, TSPAN14* | loin muscle area | MARC0113429 (87.97) | ASGA0064744 (T) - MARC0113429 (A) |
| 16 | 38 - 38.99 | ALGA0090328 - ALGA0090372 | 0.52 | 0.332 | *MAP3K1;C5ORF35;MIER3;GPBP1;snoRNA;pseudogene;* | *DDX4, ANKRD55, IL6ST, IL31RA, U6, MiRNA* | *No annotated genes* | Back fat above loin muscle dorsi | ALGA0090328 (38.00); ASGA0073091 (38.09); ALGA0090355 (38.74) | - |
| 16 | 26.02 - 26.90 | MARC0009418 - MARC0031113 | 0.49 | 0.281 | *U2; 5S - rRNA; PRKAA1* | *OSMR, RICTOR, CH242-278B18.2, DAB2, C9, U6, MiRNA, U4* | *HEATR7B2, C7, C6, PLCXD3, OXCT1* | Back fat above loin muscle dorsi | - | - |
| 2 | 103.01 - 103.74 | CASI0005590 - DRGA0003221 | 0.48 | 0.315 | *No annotated genes* | *MiRNA, 7SK* | *NR2F1, FAM172A, KIAA0825, MiRNA* | Average daily gain, feed intake, body weight and loin muscle area | - | - |
| 2 | 150.07 – 151.00 | M1GA0024073 - ALGA0108863 | 0.47 | 0.395 | *SPRY4;U5;FGF1;SNORA36;ARHGAP26;BTF3;* | *FCHSD1, ARAP3, PCDH1, KIAA0141, PCDH12, RNF14, GNPDA1, NDFIP1, SPRY4, No protein product, PCDHB11, PCDHB15, TAF7, PCDHGA1, PCDHGA2, PCDHGA3, PCDHGA5, PCDHGA6,PCDHGA7, PCDHGB4, PCDHGA9, PCDHGB6, PCDHGA10, PCDHGB7, PCDHGA12, PCDHGC5, DIAPH1, HDAC3, RELL2* | *NR3C1* | Average daily gain and back fat at last rib | - | - |
| 16 | 9.06 – 10.00 | ASGA0072214 - H3GA0045996 | 0.47 | 0.341 | *No annotated genes* | *CDH18* | *ERVK-6, CDH12* | Back fat above muscle area | - | - |
| 17 | 36.01 - 37 | ASGA0076373 - H3GA0048576 | 0.44 | 0.414 | *PANK2;miRNA;MAVS;C20ORF29;CDC25B;CENPB;SPEF1;C20ORF27;HSPA12B;SIGLEC1;ADAM33;GFRA4;ATRN;U6;C20ORF194;novel protein coding;SLC4A11;ITPA;DDRGK1;FASTKD5;UBOX5;AVP;OXT;* | *Psuedogene, NANP, NINL, GINS1, ADRA1D, SMOX, U6* | *VPS16, PCED1A, TMEM239, CPXM1, EBF4, TMC2, NOP56, IDH3B, SNRPN, TGM3, STK35, SIRPB1, CH242-165N9.3,SIRPD, CH242-240D14.1, MRPS26, PTPRA, SNORD57, SNORD56, SNORD86, SNORD64, SNORD110, SNORA51* | Average back fat thickness | - | - |
| 1 | 55.01 - 55.97 | ALGA0003197 - ASGA0002688 | 0.43 | 0.295 | *snRNA;BAI3;miRNA;* | *BAI3, U6* | *LMBRD1, COL19A1, COL9A1, FAM135A* | Average daily gain, feed intake, body weight and loin muscle area | ALGA0003197 (55.01), ALGA0003200 (55.04), ALGA0003201 (55.06), ALGA0003202 (55.08), ASGA0002670 (55.19), H3GA0001640 (55.22), MARC0051272 (55.35), H3GA0001645 (55.50), ASGA0002675 (55.55), ALGA0003221 (55.93) | ALGA0003197 (C) - ALGA0003200 (G) - ALGA0003201 (T) - ALGA0003202 (T) - ALGA0109305 (G) - ASGA0002670 (A) - H3GA0001640 (C) - DRGA0000832 (C) - DRGA0000833 (G) |
| 1 | 56.00- 56.99 | MARC0094661 - ALGA0112981 | 0.43 | 0.306 | *LMBRD1;COL19A1;COL9A1;FAM135A;* | *BAI3, MiRNA, U6* | *Pseudogene, FAM135A, C6orf57, SMAP1, B3GAT2, OGFRL1, SNORA70, ssc-mir-30c-2, ssc-mir-30a, MiRNA* | Average daily gain, feed intake, body weight and loin muscle area | ASGA0002701 (56.21), DRGA0000851 (56.29) | - |
| 12 | 25.01 - 25.98 | ASGA0085715 - ALGA0065740 | 0.39 | 0.344 | *CALCOCO2;snoU89;ATP5G1;UBE2Z;SNORA11;SNF8;GIP;novel protein coding;miRNA;B4GALNT2;ABI3;PHOSPHO1;ZNF652;PHB;NGFR;NXPH3;SPOP;SLC35B1;FAM117A;MYST2;* | *NFE2L1, CDK5RAP3, SP2, SKAP1, SNX11, CBX1, COPZ2, NFE2L1, Stat5b, STAT5A, GHDC, HOXB1, HOXB2, HOXB3, HOXB4, HOXB9, HOXB8, HOXB7, HOXB6, HOXB5, HOXB13, TTLL6, CALCOCO2, ssc-mir-152, ssc-mir-196a-1, ssc-mir-10a* | *CH242-301O20, CH242-301O20, MYST2, TAC4, TGM3, DLX3, ITGA3, PDK2, SAMD14, PPP1R9B, SGCA, HILS1, COL1A1, TMEM92, LRRC59, EME1, MRPL27, XYLT2, Lrrc37a, LRRC37B, RDM1, MRPL27, LRRC59, ACSF2, CHAD, RSAD1, EPN3, SPATA20, MYCBPAP, U6* | loin muscle area | - | - |
| 12 | 41.05 - 41.56 | H3GA0055128 - MARC0051533 | 0.38 | 0.243 | *CCL4;RPL12;CCL3L1;pseudogene;protein - coding;CCL16;CCL5;C17ORF66;TAF15;PEX12;snoZ30;miRNA;UNC45B;NLE1;FNDC8;* | *HEATR6, CCL4, LHX1, AATF, ACACA, C17orf78, TADA2A, DUSP14, SYNRG, DDX52, HNF1B, U6* | *FNDC8, RAD51D, RFFL, LIG3, CCT6B, ZNF830, Tmem132e, CCL11, CCL8, CCL1, CCL2, SNORA70, U6* | loin muscle area | ALGA0117499 (41.09) | - |
| 13 | 25.04 - 25.92 | ALGA0116157 - ALGA0123031 | 0.38 | 0.349 | *DLEC1;miRNA;OXSR1;ACAA;MYD88;protein - coding;XYLB;SLC22A14;SLC22A13;ACVR2B;EXOG;SCN5A;SCN10A;SCN11A;* | *CTDSPL, GOLGA4, ITGA9, DLEC1, PLCD1, VILL, MiRNA* | *WDR48, GORASP1, TTC21A, CSRNP1, XIRP1, CX3CR1, CCR8, SLC25A38, CASP15, RPSA, MOBP, MYRIP, SNORA62* | Loin muscle depth | - | - |
| 14 | 10.01 - 10.99 | ALGA0074961 - ALGA0075075 | 0.38 | 0.336 | *NEFL;NEFM;DOCK5;LHRH;KCTD9;protein - coding;CDCA2;EBF2;* | *ADAM28, ADAMDEC1, ADAM7* | *PNMA2, DPYSL2, ADRA1A, LOC100351641, PPP2R2A, BNIP3L* | loin muscle area | - | - |
| 5 | 11.03 - 11.94 | ALGA0030513 - ALGA0030559 | 0.37 | 0.268 | *SNORA50;U6;novel protein coding;* | *No annotated genes* | *BTBD11, PRDM7, TIMP3, SYN3, FBXO7, BPIL2, C22orf28, ASCL4, PRDM4, PWP1* | Average daily gain, body weight and back fat at loin muscle | MARC0056846 (11.41) | - |
| 18 | 31.02 – 32.00 | MARC0005136 - MARC0107657 | 0.37 | 0.339 | *ST7;CAPZA2;CAPZA;MET;CAV1;CAV2;TES;* | *CTTNBP2, CFTR, ASZ1, ST7, WNT2, ASZ1* | *TES, TFEC, U6* | Back fat and feed conversion ratio | - | - |
| 5 | 4.09 - 4.90 | M1GA0007314 - MARC0022322 | 0.36 | 0.36 | *NHP2L1, PMM1, CSDC2, XRCC6, POLR3H, PPPDE2, ACO2, L3MBTL2, CHADL, RANGAP1, EP300, XPNPEP3, ST13, SLC25A17, RPL31* | *CYB5R3, SCUBE1, TTLL12, TSPO, MCAT, SEPT3, WBP2NL, TTLL1, NAGA, FAM109B, LOC100510960, PACSIN2, NDUFA6, CYP2D6, ARFGAP3, POLDIP3, A4GALT, U12, U6* | *MCHR1, MKL1, SGSM3, ADSL, TNRC6B, FAM83F, GRAP2, ENTHD1, CACNA1I, snoU13, MiRNA* | Feed intake and back fat | - | - |
| 10 | 72.25 - 72.95 | DRGA0017455 - ASGA0106280 | 0.36 | 0.287 | *No annotated genes* | *Psuedogene, AKR1E2, MCTS1, GDI2, FAM208B, ASB13, CALML, NET1, Ucn3, TUBAL3, AKR, AKR1C4, AKR1C1,U6* | *KLF6, PFKP, PITRM1, U6* | Age at puberty | ASGA0093280 (72.90) |  |
| 10 | 15.13 - 15.99 | ALGA0057279 - H3GA0029351 | 0.33 | 0.286 | *WDR26;CNIH3;U3;protein - coding;MIXL1;ACBD3;H3F3A;C1ORF55;LEFTY2;snRNA;PYCR2;TMEM63A;* | *RGS7, FH, KMO, ZNF596, NVL, DEGS1, CNIH4, U6* | *TMEM63A, EPHX1, SRP9, ENAH, LIN9, PARP1, C1orf95, ITPKB, PSEN2, CDC42BPA* | Average daily gain, average daily lean meat gain, body weight, loin and neck meat weight | - | - |
| 17 | 65.11 - 66 | MARC0114511 - ALGA0096466 | 0.33 | 0.301 | *ZBP1;TMEPAI;C17H20ORF85;C17H20ORF86;PPP4R1L;RAB22A;VAPB;APCDD1L;* | *CR956640.5, CH242-255C19.2, CH242-266P8.1, CH242-37G9.1, CASS4, C20orf43, GCNT7, C17H20orf106, TFAP2C, BMP7, SPO11, RAE1, RNPC1, CTCFL, PCK1, U6* | *STX16, NPEPL1, GNAS, TH1L, CTSZ, TUBB1, ATP5E, SLMO2, ZNF831, EDN3, ssc-mir-296* | Back fat at last rib | M1GA0022475 (65.17) | - |
| 10 | 70.02 - 70.9 | ALGA0059917 - MARC0030594 | 0.32 | 0.304 | *protein - coding;SFMBT2;U6;miRNA;PRKCQ;PFKFB3;* | *Psuedogene, SFMBT2, GATA3, TAF3, ITIH2* | *Pseudogene, RBM17, IL2RA, IL15RA, FBXO18, ANKRD16, GDI2, FAM208B, ASB13, CALML, NET1, Ucn3, TUBAL3, AKR, AKR1C4, AKR1C1, U6* | Meat quality traits | ALGA0059997 (70.47) | - |
| 6 | 121.12 - 121.99 | MARC0012152 - MARC0009864 | 0.31 | 0.307 | *LPHN2;* | *TTLL7, U6* | *LPHN2* | Average daily gain, body weight, loin muscle area | - | - |
| 1 | 53.02 - 53.98 | ALGA0003122 - DRGA0000811 | 0.3 | 0.237 | *No annotated genes* | *EYS, U6* | *U6* | Average daily gain, feed intake, body weight, loin muscle area | ALGA0003126 (53.04), ASGA0002640 (53.67), DRGA0000805 (53.75), ASGA0002642 (53.87), ALGA0003151 (53.89), ALGA0003152 (53.91), ALGA0003154 (53.92), DRGA0000808 (53.94), ALGA0003158 (53.96), DRGA0000811 (53.97) | - |
| 15 | 131.08 – 132.00 | ASGA0092785 - MARC0070665 | 0.29 | 0.319 | *MREG;PECR;TMEM169;XRCC5;MARCH4;protein - coding;IGFBP-5;TNP1;* | *ATIC, FN1-201, MREG-201, 5S_rRNA* | *No annotated genes* | loin muscle depth | - | - |
| 2 | 98 - 99 | INRA0009055 - ALGA0014534 | 0.28 | 0.167 | *snRNA;TMEM161B;7SK;ssc-mir-9-2;MEF2C;* | *RASA1, CCNH* | *No annotated genes* | Average daily gain, body weight and loin muscle depth | ASGA0010954 (98.06), MARC0014712 (98.47) | - |
| 10 | 14.02 - 14.94 | CASI0007815 - ALGA0117764 | 0.27 | 0.294 | *WDR64;CHML;YWHAZ;novel protein - coding;U6;GREM2;RGS7;FH;KMO* | *HHIPL2, TAF1A, MIA3, AIDA, BROX, FAM177B, Psuedogene, WDR64, U6atac* | *SDE2, LEFTY2, ZNF596, NVL, DEGS1, CNIH4, WDR26, CNIH3, LIN9, MIXL1, ACBD3, H3F3A, U6, U3* | Average daily gain, average daily lean meat gain, body weight, loin and neck meat weight | - | - |
| 17 | 57.04 - 57.94 | ASGA0077474 - ASGA0077537 | 0.27 | 0.314 | *ARFGEF2;CSE1L;protein - coding;STAU1;KCNB1;PTGIS;pseudogene;B4GALT5;SLC9A8;TMEM189;UBE2V1;CEBPB;* | *PREX1, ARFGEF2* | *CH242-7P5.3, CEBPB, PTPN1, FAM65C, PARD6B, BCAS4, ADNP, DPM1, MOCS3, KCNG1* | Back fat at last rib | - | - |
| 15 | 152.02 - 152.81 | MARC0043024 - MARC0090319 | 0.24 | 0.214 | *SCLy, ESPNL, KLHL30, FAM132B, HES6, PER2, ASB1, TRAF3IP1, TWIST2, TRAF3IP1* | *COPS8, COL6A3, RAB17, Lrrfip1, RBM44, RAMP1, UBE2F* | *RANBP21, exportin-5, MRPS18A, MAD2L1BP, RSPH9, NDUFA10, HDAC4, POLH, GTPBP2, SNORA27* | Gestation length | ASGA0071782 (152.17), MARC0110475 (152.24) | H3GA0052562 (C) - M1GA0020786 (G) - CADI0000752 (C) |
| 16 | 29.06 - 29.95 | ALGA0108093 - H3GA0046366 | 0.24 | 0.25 | *C5ORF39, ZNF131, CCL28, HMGCS1, PAIP1, C5ORF34, NNT* | *GHR, CCDC152, SEPP1* | *FGF10* | Backfat at loin muscle, feed convertion ratio, daily feed intake | - | - |
| 4 | 124.04 - 124.98 | INRA0016870 - ALGA0028434 | 0.23 | 0.263 | *No annotated genes* | *NTNG1, Pesudogene, U6* | No *annotated* *genes* | Loin muscle area | INRA0016870 (124.04) | - |
| 5 | 10.08 - 10.95 | ASGA0024360 - ASGA0024449 | 0.23 | 0.253 | *No annotated genes* | *SSC.16321, APOL3, RBFOX2, APOL6, ISX, SNORA70* | *LARGE, U6, SNORA76* | Back fat above muscle dorsi, mofibril fragmentation index | H3GA0015652 (10.70) | - |
| 9 | 150.14 - 150.98 | MARC0081996 - ASGA0045362 | 0.23 | 0.241 | *AADC, DDC, GRB10, COBL* | *VWC2, ZPBP, C7orf72, IKZF1, FIGNL1, AADC, DDC, 5S_rRNA* | *No annoated gene, U6* | Diameter of tpeIIA muscle fiber | - | - |
| 16 | 36.03 - 36.93 | ASGA0073029 - MARC0027943 | 0.23 | 0.24 | *ESM1, GZMK, GZMA, CCNO, GPX8, DHX29, SKIV2L2, PPAP2A, SLC3BA9* | *ARL15, HSPB3, SNX18* | *SLC38A9, DDX4, LOC100626422, ANKRD55, IL6ST, IL31RA, U6, MiRNA* | Back fat above muscle dorsi and total bod fat | ASGA0073029 (36.02), ASGA0073030 (36.05), ALGA0090242 (36.11), ASGA0073040 (36.13) | - |
| 8 | 19.01 - 19.97 | ASGA0099602 - ASGA0038111 | 0.22 | 0.256 | *CCDC149, NP - 001072156.1, NPT2B* | *DHX15* | *SEL1L3, RBPJ, CCKAR, TBC1D19, SEL1L3, U6, U3* | Loin muscle area | ALGA0046838 (19.90) | ALGA0046838 (A) - ASGA0038101 (G) |
| 13 | 24.02 - 24.93 | ALGA0103510 - MARC0056939 | 0.22 | 0.248 | *GOLGA4, PLCD1, VILL, CTDSL* | *STAC, MLH1, No protein product, Pseudogene, LRRFIP2, EPM2AIP1, TRANK1, DCLK3, GOLGA4* | *SCN11A, CTDSPL, DLEC1, OXSR1, ACAA1, MyD88, SLC22A13, XYLB, SLC22A14, ACVR2B, EXOG, SCN5A, SCN10A, MiRNAS* | Loin muscle depth | - | - |
| 13 | 58.01 - 58.98 | ALGA0070187 - ALGA0070249 | 0.22 | 0.248 | *FOXP1, EIF4E3, PROK2* | *CU468856, FOXP1, FOXG1* | *RYBP, SHQ1, GXYLT2, U6* | Loin weight | - | - |
| 16 | 30.08 - 30.97 | ALGA0089960 - MARC0112978 | 0.22 | 0.196 | *FGF10, HCN1* | *ANXA2R, ZNF131, CCL28, HMGCS1, NIM1, C5orf28, C2H5orf28, C20H5orf34, PAIP1, C4H5orf34, NNT, MiRNA, U6* | *HCN1, ATP5H, EMB, PARP8, SNORD28* | Back fat above muscle dorsi | - | - |
| 8 | 73.04 - 73.93 | MARC0071875 - ALGA0048095 | 0.21 | 0.288 | *COX18, ANKRD17, ALB, AFP, AFM, RASSF6* | *NPFFR2, ADAMTSL1, ADAMTS3, SLC4A4, GC, U6, SNORD112* | *IL8, CBG18082, AMCF-II, CXCL7, PF4, CXCL2, MTHFD2L, EREG, SSC.82438, BTC, 7SK, 5S_rRNA* | Loin muscle area | - | - |
| 7 | 109.05 - 109.98 | H3GA0022804 - H3GA0022828 | 0.2 | 0.184 | *DIO2, CEP128, GTF2A1* | *NRXN3* | *GTF2A1, STON2, SEL1L, SNORA79, MiRNA* | Loin muscle area, depth and weight. | - | - |

^@^ The 1Mb windows are presented in descending order based on the percent genetic variance explained greater than 0.2%.

*Posterior probability that the SNPs in 1Mb window could explain the genetic variance greater than zero.

**Association of single SNPs was considered based on genomic control corrected P-values at a threshold of 0.01 by the PLINK software

***Association of haplotypes was considered based on genomic control corrected P-values at a threshold of 0.05 by the PLINK software

^$^ The genes and their abbreviations are based on *Sus scrofa* genome build 10.2

Note: The windows with unmapped SNPs are not real consecutive SNP windows and hence they are not presented
